# Genome-wide CRISPR screen reveals v-ATPase as a drug target to lower levels of ALS protein ataxin-2

**DOI:** 10.1016/j.celrep.2022.111508

**Published:** 2022-10-25

**Authors:** Garam Kim, Lisa Nakayama, Jacob A. Blum, Tetsuya Akiyama, Steven Boeynaems, Meenakshi Chakraborty, Julien Couthouis, Eduardo Tassoni-Tsuchida, Caitlin M. Rodriguez, Michael C. Bassik, Aaron D. Gitler

**Affiliations:** 1 Department of Genetics, Stanford University School of Medicine, Stanford, CA 94305, USA; 2 Stanford Neurosciences Interdepartmental Program, Stanford University School of Medicine, Stanford, CA 94305, USA; 3 Department of Biology, Stanford University School of Medicine, Stanford, CA 94305, USA; 4 Lead contact

## Abstract

Mutations in the ataxin-2 gene (*ATXN2*) cause the neurodegenerative disorders amyotrophic lateral sclerosis (ALS) and spinocerebellar ataxia type 2 (SCA2). A therapeutic strategy using antisense oligonucleotides targeting *ATXN2* has entered clinical trial in humans. Additional ways to decrease ataxin-2 levels could lead to cheaper or less invasive therapies and elucidate how ataxin-2 is normally regulated. Here, we perform a genome-wide fluorescence-activated cell sorting (FACS)-based CRISPR-Cas9 screen in human cells and identify genes encoding components of the lysosomal vacuolar ATPase (v-ATPase) as modifiers of endogenous ataxin-2 protein levels. Multiple FDA-approved small molecule v-ATPase inhibitors lower ataxin-2 protein levels in mouse and human neurons, and oral administration of at least one of these drugs—etidronate—is sufficient to decrease ataxin-2 in the brains of mice. Together, we propose v-ATPase as a drug target for ALS and SCA2 and demonstrate the value of FACS-based screens in identifying genetic—and potentially druggable—modifiers of human disease proteins.

## In brief

Kim et al. perform a genome-wide CRISPR knockout screen for modifiers of levels of the ALS and SCA2 protein ataxin-2. Genetic and pharmacologic inhibition of the lysosomal v-ATPase decreases ataxin-2 protein levels *in vitro*. Oral administration of an FDA-approved small molecule v-ATPase inhibitor is sufficient to lower ataxin-2 protein levels in the brains of mice.

## Graphical Abstract

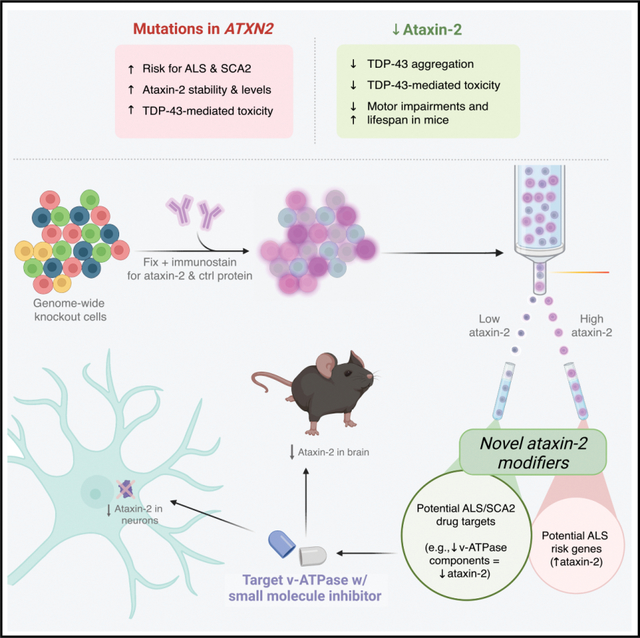

## INTRODUCTION

Many neurodegenerative diseases are associated with accumulation of misfolded proteins (Forman et al., 2004), and strategies to rid these proteins are emerging as a promising therapeutic approach (Bennett et al., 2021). Hyperphosphorylated TDP-43 aggregates are present in the brains of nearly all (~97%) patients with amyotrophic lateral sclerosis (ALS) (Neumann et al., 2006) as well as in numerous other neurodegenerative disorders (de Boer et al., 2020). Despite TDP-43 pathology being a common feature in ALS, the genetic causes underlying the disorder are numerous and wide ranging (Kim et al., 2020). In this context, ataxin-2—encoded by the gene *ATXN2*—has emerged as a therapeutic target because it is a potent genetic modifier of TDP-43 toxicity and aggregation. Increased levels of ataxin-2 exacerbate TDP-43-induced toxicity, while decreased ataxin-2 levels mitigate the toxicity (Armakola et al., 2011; Becker et al., 2017; Elden et al., 2010; Kim et al., 2014).

Ataxin-2 is a polyglutamine (polyQ) protein in which long (>34) polyQ expansions cause spinocerebellar ataxia 2 (SCA2) (Imbert et al., 1996; Pulst et al., 1996; Sanpei et al., 1996), and intermediate-length (27–33) repeats are a risk factor for ALS (Elden et al., 2010). Antisense oligonucleotides (ASOs) targeting ataxin-2 *in vivo* provide marked protection against motor deficits and extend lifespan in mouse models of TDP-43 proteinopathy (Becker et al., 2017) and SCA2 (Scoles et al., 2017). These results have motivated recent administration of ASOs targeting *ATXN2* to human patients with ALS with or without polyQ expansions in a phase 1 clinical trial (ClinicalTrials.gov: NCT04494256). Gene-based therapies, like ASOs, hold great promise to provide disease-modifying treatments for these devastating neurodegenerative diseases but are yet unproven for patients with ALS. Despite this enormous progress, questions remain regarding the potential safety or dosage limitations of using ASOs, and little is known about how ataxin-2 is normally regulated. Thus, we searched for other ways to modulate ataxin-2 protein levels.

We developed a fluorescence-activated cell sorting (FACS)-based screening method using pooled CRISPR-Cas9-mediated genome-wide deletion libraries in conjunction with antibody staining to detect endogenous protein levels. The screen revealed numerous previously unknown genetic modifiers of ataxin-2 protein levels, including multiple subunits of the lysosomal vacuolar ATPase (v-ATPase). We demonstrate that inhibiting the lysosomal v-ATPase with small-molecule drugs (FDA approved) results in decreased ataxin-2 protein levels in human and mouse neurons, as well as *in vivo* in the brains of mice upon oral administration of one of the drugs—etidronate. These results exemplify the value and ease (screen from start to finish in 1 week) of a FACS-based screening approach in defining regulators of normal and/or disease proteins and the potential to repurpose readily available small-molecule drugs for diseases impacted by protein levels.

## RESULTS

### FACS-based genome-wide CRISPR-Cas9 KO screens in human cells reveal modifiers of ataxin-2 protein levels

**Figure 1.**
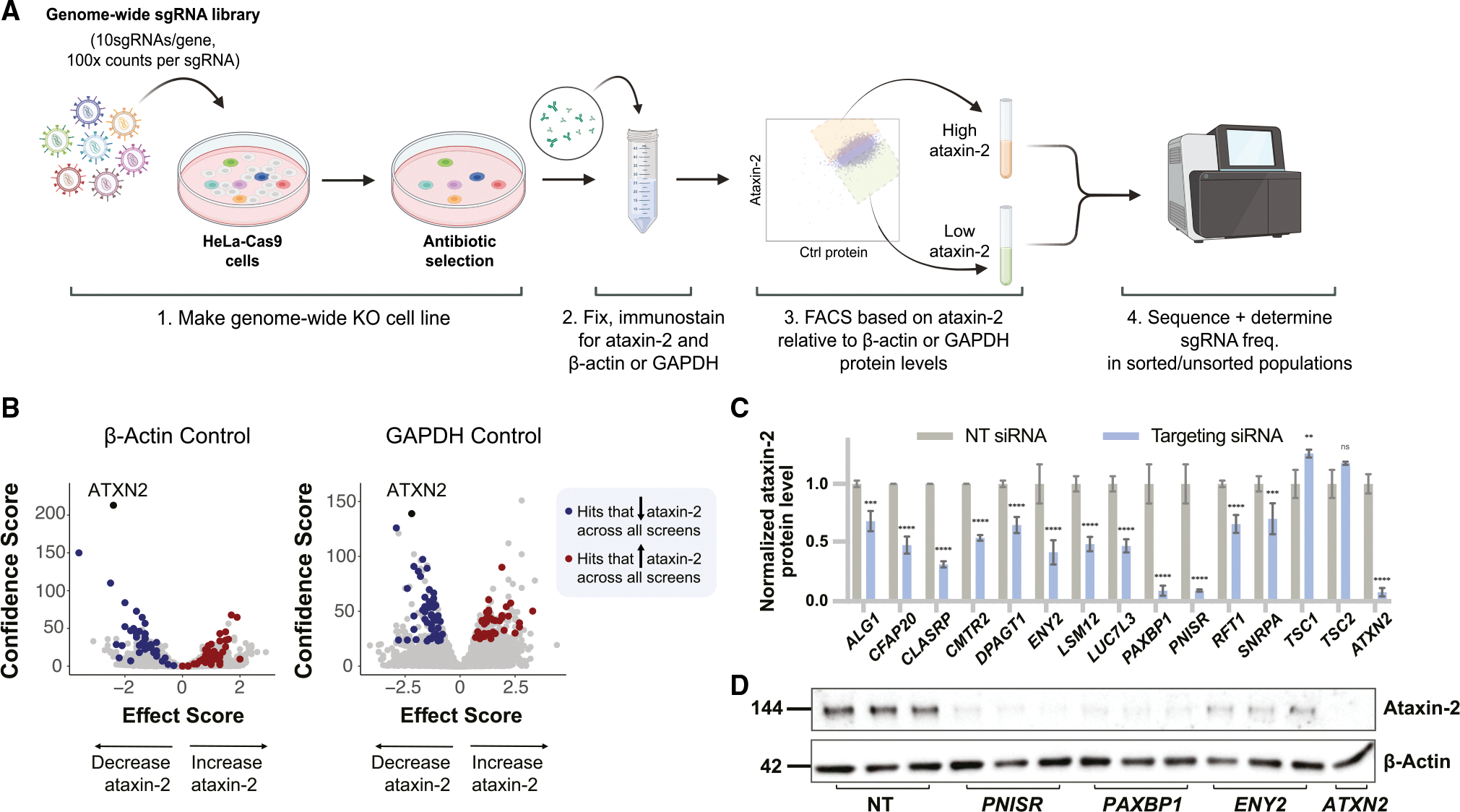
Genome-wide CRISPR-Cas9 KO screens in human cells identify regulators of ataxin-2 protein levels (A) Pooled CRISPR-Cas9 screening paradigm. After transducing HeLa cells expressing Cas9 with a lentiviral whole-genome sgRNA library, we fixed and co-immunostained the cells for ataxin-2 and a control protein (β-actin or GAPDH). We then used FACS to sort top and bottom 20% ataxin-2 expressors relative to control protein levels (duplicate sorts per each control). After isolating genomic DNA from these populations, as well as the unsorted control population, we performed NGS to read sgRNA barcodes. (B) Volcano plots based on effect and confidence scores summarizing genes that modify ataxin-2 protein levels relative to β-actin (left) or GAPDH (right) levels when knocked out (FDR <0.05). Highlighted in blue and red are hits that overlap across all screens. (C) Validation of numerous top hit genes overlapping across all screens. We transfected HeLa-Cas9 cells with non-targeting (NT) siRNAs or with siRNAs targeting mRNA transcripts encoded by hit genes, then performed immunoblotting on lysates. Quantifications are normalized to the NT siRNA condition (mean ± SD; analyzed using two-way ANOVA; ****p < 0.0001, ***p < 0.001, **p < 0.01, *p < 0.05, ns: not significant). (D) Representative immunoblot of ataxin-2 and β-actin protein levels upon treatment with NT, *PNISR*, *PAXBP1*, or *ATXN2* siRNAs in HeLa cells.

To find additional ways (e.g., new targets or pathways) to lower ataxin-2 levels, we developed a FACS-based genome-wide CRISPR knockout (KO) screen for modifiers of endogenous ataxin-2 protein levels (i.e., without protein tags like Flag or GFP or overexpression) (Figure 1A). We optimized conditions to sensitively detect changes in ataxin-2 protein levels in human cells by antibody staining and FACS (Figure S1) and engineered HeLa cells to stably express Cas9 along with either a blasticidin-resistance cassette (HeLa-Cas9-Blast) or blue fluorescent protein (HeLa-Cas9-BFP). To create genome-wide KO cell lines, we transduced HeLa-Cas9-Blast and HeLa-Cas9-BFP cells (as biological replicates) with a lentiviral single guide RNA (sgRNA) library comprising 10 sgRNAs per gene—targeting ~21,000 human genes—and ~10,000 safe-targeting sgRNAs (Morgens et al., 2017) (Figure 1A). After fixing the cells in methanol and immunostaining them with antibodies targeting endogenous ataxin-2 and a control protein (GAPDH or β-actin), we used FACS to sort the highest and lowest 20% levels of endogenous ataxin-2 expressors relative to levels of the control protein (Figure 1A). We performed the screen four times, twice each with β-actin and GAPDH as the control proteins. We used two different control proteins instead of one to minimize the possibility of selecting hits that were simply global regulators of transcription, translation, or cell size or otherwise idiosyncratic to the biology of a given control protein.

After sorting cells based on ataxin-2 expression levels, we extracted genomic DNA and performed next-generation sequencing (NGS) of the barcoded sgRNAs. Using the Cas9 high-throughput maximum-likelihood estimator (casTLE) algorithm (Morgens et al., 2016), we isolated genes that, when knocked out, increased or decreased ataxin-2 protein levels (Figure 1B). A false discovery rate (FDR) cutoff of 5% revealed an overlapping set of 52 gene KOs that decreased—and 36 that increased—ataxin-2 levels across the four screens. As expected, the strongest hit that lowered ataxin-2 levels was *ATXN2* itself, demonstrating the effectiveness of this screening approach (Figure 1B). The full list of hits from the screens is presented in Table S1.

**Figure 2.**
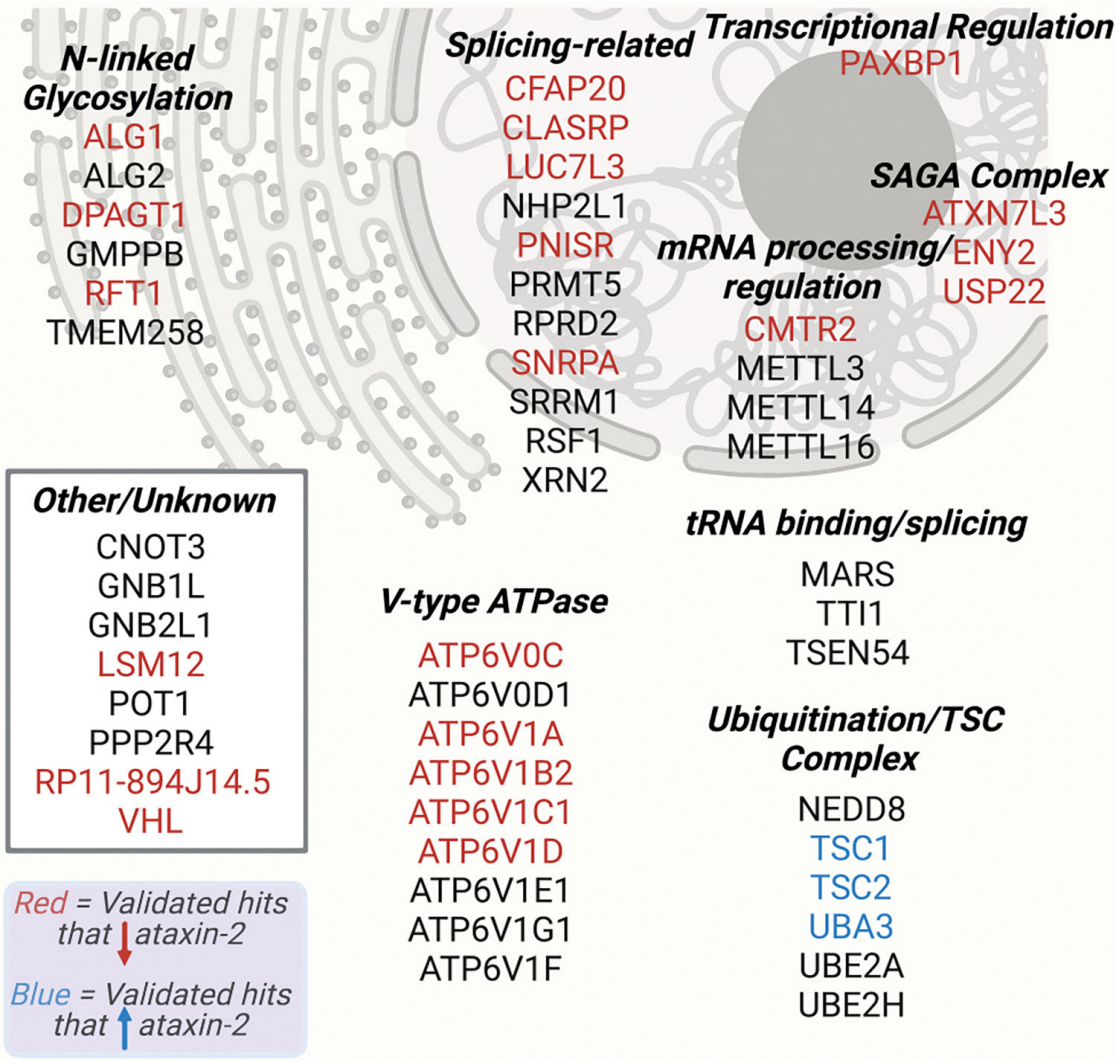
Schematic of proteins encoded by selected hits (5% FDR), categorized by function and subcellular localization

We individually validated screen hits by treating HeLa cells with siRNAs targeting those gene products followed by immunoblotting. We selected genes for follow up based on a combination of significance (casTLE) score and effect size (Morgens et al., 2016) (Table S1), as well as known and predicted direct (physical) and indirect (functional) associations with ataxin-2 or between hits. The list of hits included genes known to have a direct association with ataxin-2, such as *LSM12*, as well as many previously unknown and potent genetic modifiers of ataxin-2 protein levels, such as *CFAP20*, *CLASRP*, *CMTR2*, *LUC7L3*, *PAXBP1*, *PNISR*, *ATP6V1A*, and *ATP6V1C1*, and others (Figures 1C, 1D, and 2). Strikingly, some of the hits (e.g., *PNISR* and *PAXBP1*) lowered ataxin-2 levels as much as targeting *ATXN2* itself (Figure 1D). We further validated hits in the human neuroblastoma cell line SH-SY5Y, where many—but not all—had a significant effect on ataxin-2 protein levels (Figures S2A and S2B). In addition to identifying numerous previously unknown regulators of ataxin-2, the high rate of hit validation from the initial screen and its relative ease (a genome-wide screen can be completed in <1 week from cell culture to NGS analysis) suggests that these FACS/antibody-staining based screens—using fixed cells to uncover regulators of endogenous protein levels—may be useful in many other contexts.

### Inhibiting lysosomal v-ATPase via genetic or pharmacologic perturbation lowers ataxin-2 protein levels and stress granule number *in vitro*

**Figure 3.**
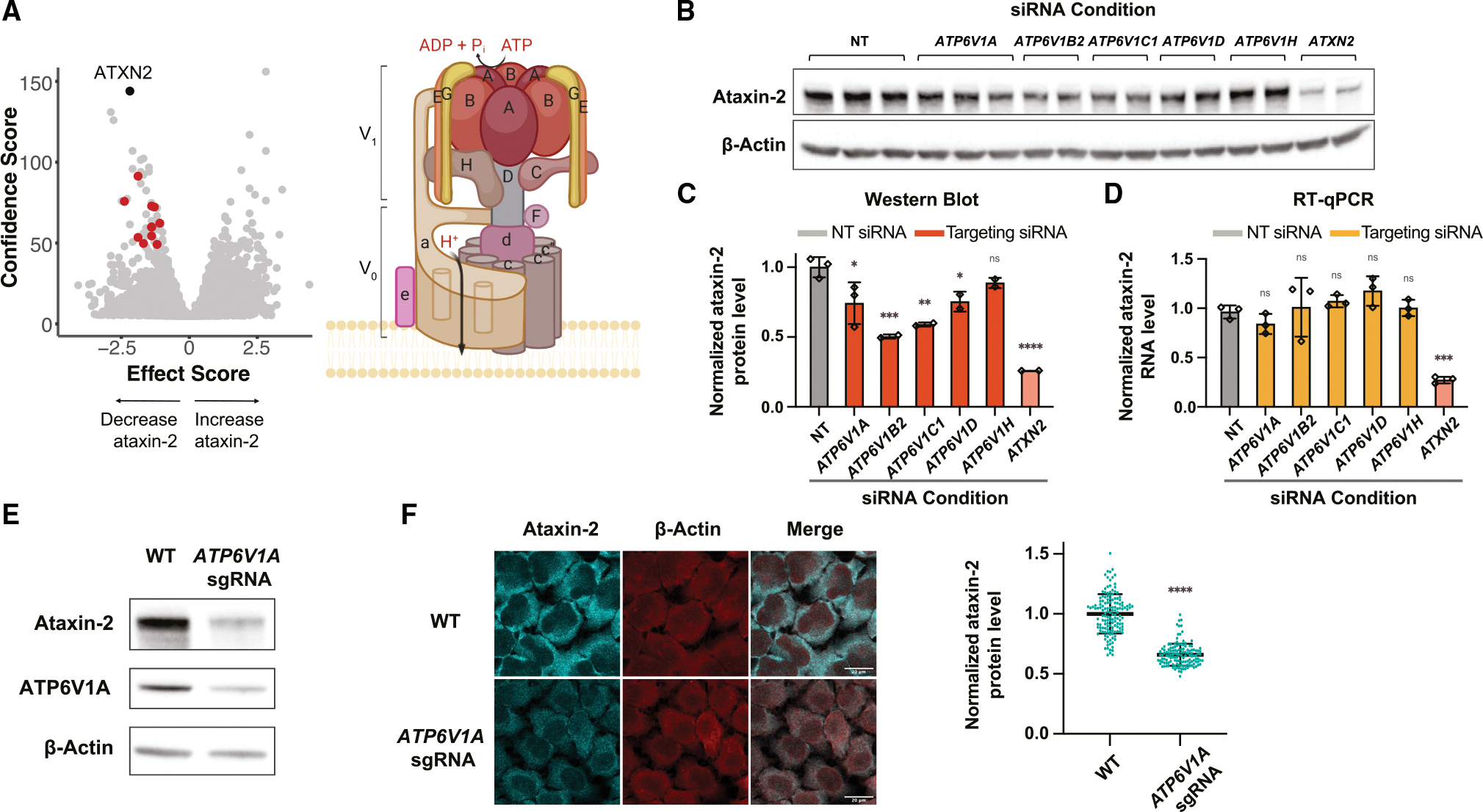
Genetically and pharmacologically perturbing lysosomal v-ATPase leads to decreased ataxin-2 protein levels *in vitro* (A) Left, volcano plot shows confidence score on y axis and effect score on x axis, with gene hits encoding lysosomal v-ATPase subunits highlighted in red. Right, a representation of the lysosomal v-ATPase, with its V_0_ and V_1_ domains, as well as individual subunits. (B and C) Immunoblot (B) and quantification (C) of ataxin-2 protein levels after HeLa cells were transfected with siRNAs against various v-ATPase subunits. (D) Quantification of *ATXN2* RNA levels after siRNA knockdown of v-ATPase subunits in HeLa cells. Values normalized to β-actin RNA levels. Quantifications for (C) and (D) are normalized to the NT siRNA condition (mean ± SD; analyzed using one-way ANOVA with post hoc Dunnett’s multiple comparisons tests; ****p < 0.0001, ***p < 0.001, **p < 0.01, *p < 0.05, ns: not significant). (E) Immunoblot on lysates from WT or *ATP6V1A* Cas9-edited HeLa cell lines. (F) Representative microscopy images of WT or *ATP6V1A* Cas9-edited HeLa cells, immunostained for ataxin-2 or β-actin (scale bar: 20 μm). Ataxin-2 fluorescence quantifications are shown on the right (lines denote mean ± SD; analyzed using unpaired t test; ****p < 0.0001).

We observed a striking number of genes encoding subunits of lysosomal v-ATPases that decreased ataxin-2 levels when knocked out (Figure 3A). Lysosomal v-ATPases help to maintain an acidic pH (~4.5) in the lysosome by pumping protons from the cytosol to the lumen via consumption of ATP (Maxson and Grinstein, 2014). We used siRNAs and immunoblotting to confirm that knocking down numerous v-ATPase subunits leads to decreased ataxin-2 protein levels (Figures 3B and 3C). Using CRISPR-Cas9, we also verified that constitutive downregulation of ATP6V1A—a central subunit in v-ATPase function (Maxson and Grinstein, 2014)—resulted in a marked decrease in ataxin-2 protein levels (Figures 3E and 3F). Levels of other polyQ disease proteins huntingtin and ataxin-1 were unaltered in these cells (Figures S3A and S3B), while TDP-43 was modestly decreased (Figures S3C and S3D). There remains the possibility of wider yet unexplored protein-level effects downstream of v-ATPase inhibition. To determine whether the mechanism of regulation was at the protein or RNA level, we performed RT-qPCR after applying siRNAs to knock down numerous v-ATPase subunits and observed no change in steady-state *ATXN2* mRNA levels (Figure 3D). RNA sequencing confirmed that knocking down a v-ATPase subunit using siRNAs did not lead to changes in *ATXN2* mRNA levels or other noteworthy transcriptional changes (Figure S4). These results provide evidence that the mechanism of v-ATPase regulation of ataxin-2 is post-transcriptional.

In addition to being enriched as screen hits, the v-ATPases stood out for another reason: the availability of small-molecule inhibitors. One of these small-molecule v-ATPase inhibitors is etidronate (~206 Daltons), which was FDA approved in 1977 as a drug to treat Paget disease of bone (Altman et al., 1973). Interestingly, Paget disease of bone has been connected to TDP-43 proteinopathy (Gitcho et al., 2009; Neumann et al., 2007; Watts et al., 2004). Etidronate is a bisphosphonate whose chemical structure and high affinity for bone minerals very selectively induces apoptosis in osteoclasts, popularizing their use in skeletal disorders like osteoporosis over multiple decades (Drake et al., 2008). Despite their apoptosis-inducing role in osteoclasts, bisphosphonates have also been suggested to limit apoptosis in other cell types, and their structural similarity to inorganic pyrophosphate led to the discovery that bisphosphonates inhibit the v-ATPase (David et al., 1996; Drake et al., 2008). Given etidronate’s wide therapeutic index, small size, and known safety in humans, we tested whether etidronate treatment could lower ataxin-2 levels in human and mouse neurons. We also tested whether two other FDA-approved drugs—alendronate and thonzonium—would decrease ataxin-2 protein levels. Alendronate (~249 Daltons) is a second-generation bisphosphonate (also known as Fosamax), whereas thonzonium (~511 Daltons) inhibits the v-ATPase through a different mechanism (uncoupling the proton transport and ATPase activity of the v-ATPase proton pumps).

**Figure 4.**
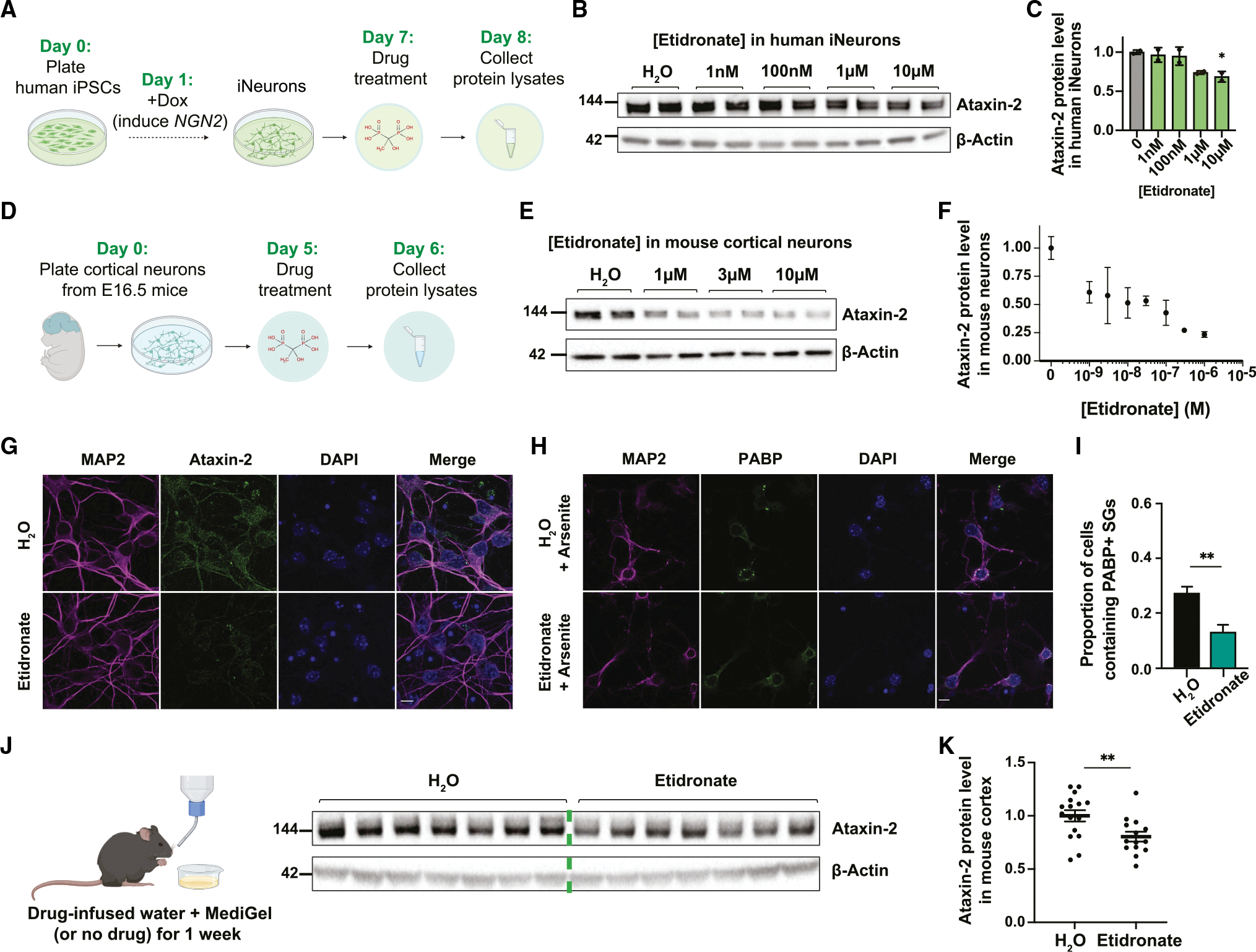
Small-molecule drug etidronate lowers ataxin-2 protein levels in human iPSC-derived neurons, mouse primary neurons, and *in vivo* in mice (A) Timeline of induced neuron differentiation in a human iPSC line with *NGN2* stably integrated and drug treatment. (B) Immunoblot on lysates from human iPSC-derived neurons treated with various doses of etidronate. (C) Quantification of Figure 4B, with ataxin-2 protein levels normalized to H_2_O-treated condition (mean ± SD; analyzed using one-way ANOVA with post hoc Dunnett’s multiple comparisons tests; *p < 0.05). (D) Timeline of primary neuron plating from embryonic mouse cortex and drug treatment. (E) Immunoblot on lysates from mouse primary neurons treated with various doses of etidronate. (F) Quantification of the dose-dependent effect of etidronate on ataxin-2 (normalized to control condition). (G) Representative microscopy images of mouse cortical neurons treated with sham (H_2_O) or 10 μM etidronate for 24 h, stained for MAP2, ataxin-2, and DAPI (scale bar: 10 μm). (H) Representative microscopy images of mouse cortical neurons treated with H_2_O or 10 μM etidronate for 24 h, with 0.5 mM sodium arsenite treatment for the final hour. The neurons were stained for PABP, MAP2, and DAPI (scale bar: 10 μm). (I) Quantifications of cells containing PABP-positive stress granules (SGs) (mean ± SEM; analyzed using unpaired t test; ****p < 0.0001, **p < 0.01). (J) Example immunoblot of cortex lysates from mice given normal or drug-infused water and MediGel. (K) Quantification of immunoblot from Figure 4J, probing for ataxin-2 (normalized to H_2_O-treated condition) using lysates from cortices of mice that received water or drug treatment (mean ± SEM; analyzed using Welch’s t test; **p < 0.01). We performed the experiment two independent times, for a total of n = 16 in the control group and n = 14 in the drug treatment group.

Treating human neurons (induced pluripotent stem cell [iPSC]-derived or neuroblastoma SH-SY5Y cells) for 24 h with etidronate, alendronate, or thonzonium resulted in dose-dependent decreases in ataxin-2 protein levels (Figures 4A–4C, S5A, S5B, S6A, and S6B). We also tested all three drugs in mouse cortical neurons *in vitro* and observed similar dose-dependent ataxin-2 decreases (Figures 4D–4G, S5C, S5D, S6C, and S6D). Although thonzonium and alendronate decreased ataxin-2 protein levels across a wide range of doses (Figures S5C, S5D, S6C, and S6D), they became toxic to mouse neurons at concentrations greater than 10 μM (data not shown). This toxic dose has been previously reported for thonzonium (Chan et al., 2012). Etidronate, however, was not toxic even at doses up to 100 μM (highest concentration tested) (Figures 4E and 4F). The differences in toxic doses align with their known differences in IC_50_ (the concentration of drug that is needed to inhibit a biological process by half) (David et al., 1996). Given etidronate’s well-known safety profile and small size, we chose to focus on etidronate for the following experiments. Still, it is noteworthy that three different v-ATPase inhibitors—working through distinct mechanisms—potently decrease ataxin-2 protein levels in neurons without causing the type of toxicity seen in osteoclasts.

We next tested etidronate’s effects on TDP-43 levels and/or localization, since TDP-43 nuclear depletion and cytoplasmic aggregation are pathologic hallmarks of ALS (Neumann et al., 2006). When we treated mouse cortical neurons with 10 μM etidronate, we saw a slight (~10%) decrease in TDP-43 levels in the nucleus and cytoplasm (Figures S7A–S7C), similar to our observation of a moderate TDP-43 decrease in *ATP6V1A* Cas9-edited HeLa cells (Figures S3C and S3D). Etidronate treatment did not, however, affect the nuclear-to-cytoplasmic TDP-43 ratio (Figure S7D). Given the protective role of ataxin-2 in formation of stress granules (cytoplasmic foci that form in response to various forms of stress) (Becker et al., 2017; Zhang et al., 2018), we also tested the effect of etidronate treatment on stress-granule formation. Specifically, we treated mouse primary neurons with 10 μM etidronate for 23 h prior to adding 0.5 mM sodium arsenite for 1 h to induce stress-granule formation. Compared with non-drug treated cells, etidronate-treated neurons had fewer stress granules (Figures 4H and 4I). Since stress-granule formation increases the likelihood of subsequent conversion into pathologic insoluble aggregates (Li et al., 2013), we hypothesize that inhibiting the v-ATPase using etidronate may reduce the propensity for pathologic aggregate formation.

### Oral administration of etidronate lowers ataxin-2 protein levels in the brains of adult mice

Given our *in vitro* findings that these v-ATPase inhibitors can lower ataxin-2 levels, we next tested if peripheral administration of etidronate *in vivo* could decrease ataxin-2 levels in the brains of mice. Because oral administration of the drug mimics the most common mode of drug intake for humans and given the wide range of doses that lowered ataxin-2 protein levels *in vitro* (Figures 4C and 4D), we dissolved etidronate into drinking water (~2 μg/mL concentration) and MediGel (ClearH_2_O) (~20 mg/kg/day concentration) and allowed voluntary consumption by wildtype adult mice (~3–4 months old) over 1 week (Figure 4J). We performed immunoblotting on cortical extracts from mice that drank and ate either normal or drug-infused water and MediGel, respectively. After a 1 week treatment period, we observed a ~20% decrease in ataxin-2 protein levels in the brains of mice that consumed etidronate compared to the control group (n = 15 in each group) (Figures 4J and 4K). These findings suggest that etidronate administration in the water supply and MediGel are sufficient to lower levels of ataxin-2 in the brains of mice.

## DISCUSSION

Here, we present a FACS-based CRISPR screen to uncover numerous regulators of ataxin-2 levels—a validated target for ALS and SCA2 based on human genetics—including genes encoding several subunits of lysosomal v-ATPases for which small-molecule drugs are available. We demonstrate that multiple small-molecule v-ATPase inhibitors can safely and effectively decrease ataxin-2 levels in mouse and human neurons across a wide range of doses *in vitro* and that etidronate can lower ataxin-2 levels in the brain *in vivo* when orally administered to mice. We also confirm that treating neurons with alendronate—a structural analog of etidronate—leads to decreased ataxin-2 levels, indicating the potential to further develop and/or test structural bisphosphonate analogs that can most effectively cross the blood-brain barrier (BBB) and decrease ataxin-2 levels in the brain. Given etidronate’s known safety profile and our demonstration of its ability to lower ataxin-2 levels in the brain *in vivo,* we postulate that etidronate or other bisphosphonates could serve as a starting point for future optimization to decrease ataxin-2 levels in the brain. While we did not test the efficacy of etidronate in lowering polyQ-expanded forms of ataxin-2, we speculate that v-ATPase inhibition may also be effective against longer polyQ expansions because previous studies have reported that longer repeat lengths enhance ataxin-2 stability, thereby increasing its levels (Elden et al., 2010; Hart and Gitler, 2012). Clinical trials in humans will be required to determine etidronate or other bisphosphonates’ safety and efficacy as a treatment for ALS or SCA2. ASO and gene therapy approaches show promise for neurodegenerative disease but are currently prohibitively expensive (hundreds of thousands of US dollars per year for ASOs or several million US dollars for a one-time gene therapy treatment). We purchased 1 g of etidronate for $50. If effective as a therapeutic, the affordability of these compounds could be especially useful in developing countries, like Cuba, where SCA2 is relatively common.

This study is accompanied by a partner manuscript (Rodriguez et al., 2022) in which we present a complementary screen for regulators of ataxin-2 using a distinct approach: a whole-genome arrayed siRNA-based screen in a HEK293T cell line containing an 11 amino acid HiBit tag in frame with ataxin-2. This screen resulted in the discovery of the NoGo-receptor gene *RTN4R* as a potent modifier of ataxin-2 levels *in vitro* and *in vivo* (Rodriguez et al., 2022). However, there was very little overlap in the hits between both screens (only one gene in common, *LSM12*) even though they were both designed to find regulators of ataxin-2 levels. Why? There are several potential explanations to consider. We performed this screen in HeLa cells and the other one in HEK293T cells. This screen was a CRISPR-Cas9-based KO screen and the other one was an siRNA knockdown screen using the HiBit reporter as a readout. However, both screens did reveal several converging themes of ataxin-2 regulation, such as the presence of many spliceosome components and LSm domain-containing proteins. Moreover, in both studies, we took special care to validate many hit genes individually in the cell lines in which the screens were conducted to ensure the robustness and validity of each respective screen. Given the high rates of hit validation for each screen, we speculate that the lack of hit overlap is largely due to differences in the cell types (i.e., different genes expressed) and screening systems (CRISPR KO versus HiBit knockdown). There are well-known differences in effects of gene knockdown versus gene KO (Rossi et al., 2015). Another contributor to differences between the two screens could stem from one screen being pooled (all cells mixed together and competing against one another) while the other is arrayed (analyzed one by one). It may also simply be that ataxin-2 is regulated very differently across cell types. Importantly, we followed up on hits of interest to confirm that their regulation of ataxin-2 was conserved in more disease-relevant neuronal cell lines, such as SH-SY5Y neuroblastoma cells, human iPSC-derived neurons, and mouse cortical neurons. The differences in hits between the different screening platforms and cell lines underscores the importance of validating hits in disease-relevant cell lines. New CRISPR-based gene perturbation libraries will empower arrayed genome-wide screens (Yin et al., 2022). Since many human diseases are caused by moderate increases or decreases in a gene product, we suspect that the protein level-based screening approach presented here, in the accompanying manuscript by Rodriguez et al., Cell Reports 2022 and in screens by others (Lu et al., 2013; Park et al., 2013; Rousseaux et al., 2018; Yin et al., 2022) could be broadly applicable to different human disease situations, including haploinsufficiency.

### Limitations of the study

Although we demonstrate that etidronate can lower levels of ataxin-2 *in vitro* and *in vivo,* a limitation of this study is that we did not test whether the drug can rescue degeneration and motor phenotypes in a mouse model. Moreover, the mechanism through which the v-ATPase regulates ataxin-2 levels remains elusive. As we continue to pursue this target and etidronate, it will be important to (1) define the effects of v-ATPase inhibition on lysosomal function (e.g., its role in processing cellular enzymes like cathepsins) and (2) define what level of v-ATPase inhibition is tolerated and if an appropriate therapeutic index can be achieved, resulting in decreased ataxin-2 levels but not impairing other important cellular functions. Despite these unknowns, etidronate’s safety profile is well known, and there is familiarity and experience with its application in the real world given its use for treatment of Paget disease of bone and osteoporosis for over 40 years. Our previous study demonstrated that lowering levels of ataxin-2 prolonged survival and ameliorated motor impairments in a mouse model of TDP-43 proteinopathy (Becker et al., 2017). We faced difficulty in testing oral administration of etidronate in this mouse model (Wils et al., 2010) because of the aggressive progression of disease (mice succumb prior to weaning age, at which point mice begin to eat/drink from their own food/water supplies). Efforts are underway to test the efficacy of this drug in ameliorating ALS phenotypes in other mouse models of ALS with later disease onset (Arnold et al., 2013); we remain encouraged by the ability of etidronate to lower ataxin-2 levels *in vivo*, as *ATXN2* is a previously validated therapeutic target in mouse models of ALS (Becker et al., 2017) and SCA2 (Scoles et al., 2017) as well as by human genetics (Elden et al., 2010; Scoles and Pulst, 2018).

## STAR★METHODS

### RESOURCE AVAILABILITY

#### Lead contact

Further information and requests for resources and reagents should be directed to and will be fulfilled by the lead contact, Aaron D. Gitler (agitler@stanford.edu).

#### Materials availability

All unique/stable reagents generated in this study are available from the lead contact.

### Data and code availability

- All sequencing data is available under Gene Expression Omnibus accession no. GSE189417.
- Source code for analyzing CRISPR screen data using casTLE method (Morgens et al., 2016) can be found at the following URL: https://bitbucket.org/dmorgens/castle/downloads/. Detailed pipelines and options used for casTLE and RNA-seq are available on https://github.com/emc2cube/Bioinformatics/ (Kramer et al., 2018).
- Any additional data supporting the findings of this study are available from the corresponding author upon reasonable request.

### EXPERIMENTAL MODEL AND SUBJECT DETAILS

#### Cell culture

HeLa and HEK293T cells (ATCC®) were cultured in DMEM, high glucose, GlutaMAX™, HEPES media (Gibco) containing 10% fetal bovine serum (FBS) (Omega) and 1% penicillin-streptomycin (P/S) (Gibco) in a controlled humidified incubator at 37°C with 5% CO_2_. SH-SY5Y cells (ATCC®) were cultured in DMEM/F12, GlutaMAX™-supplemented media (Gibco) containing 10% FBS (Omega) and 1% P/S (Gibco) at 37°C with 5% CO_2_.

#### Mouse primary cortical neurons

Mouse primary neurons were obtained from timed-pregnant, C57BL/6J mice at E16.5 (The Jackson Laboratory). The cortices were dissected out and dissociated into single-cell suspensions with a papain dissociation system (Worthington Biochemical Corporation) and plated onto poly-L-lysine (Sigma Aldrich)-coated plates (0.1% (wt/vol)) at a density of 350,000 cells per well in 24-well plates. If used for imaging, the neurons were plated on 12mm glass coverslips (Carolina Biological Supplies cat# 633,009) in 24-well plates. The neurons were grown in Neurobasal medium (Gibco) supplemented with P/S (Gibco), GlutaMAX (Invitrogen), and B-27 serum-free supplements (Gibco) at 37°C with 5% CO_2_. Half or full media changes were performed every 3 or 4 days, or as required.

#### Neuron differentiation from iPSCs

Human iPSCs-derived neurons (iNeurons) were induced utilizing a Tet-On induction system to express the transcription factor Ngn2. Briefly, iPSCs were maintained in mTeSR1 medium (Stemcell Technologies) on Matrigel-coated plates (Fisher Scientific CB-40230). The following day, doxycycline (2 μg/mL) was added to the media to induce Ngn2 expression, followed by puromycin (2 μg/mL) treatment to rapidly and efficiently induce iNeurons. Three days following induction, the differentiating iNeurons were dissociated using Accutase (STEMCELL Technologies) and resuspended in a culture medium consisting of Neurobasal media (Thermo Fisher), N2 (Thermo Fisher), B-27 (Thermo Fisher), and BDNF/GDNF (R&D Systems) on Matrigel-coated assay plates. This resuspension mixture was then plated onto Matrigel-coated 24-well plates. Half-media changes were performed every 2–3 days.

#### Mouse husbandry

All mouse experiments were approved by the Stanford University Administrative Panel on Animal Care (APLAC). All mice (up to five animals per cage) were provided food and water and maintained on a regular 12 h light-dark cycle. For cortical neuron culturing from embryonic mice, primary neurons were obtained from pregnant wild-type C57BL/6J dams at E16.5. For *in vivo* drug treatment experiments, 3- to 4-month-old C57BL/6J mice (15 males, 15 females total) were utilized.

## METHOD DETAILS

### Pooled FACS screen

#### Generating HeLa-Cas9 cells

To generate cells that stably express Cas9, lentivirus containing Cas9 with blasticidin resistance cassette (Cas9-Blast) or blue fluorescent protein (Cas9-BFP) were generated using standard protocols (third-generation packaging plasmids pMDLg-pRRE, pRSV-Rev, pMD2.G (Addgene)). Low-passage HeLa cells were transfected at 40–50% confluency in a 100 mm dish with lentiviral concentrations such that the infection rate was ~20%, to reduce the chance that a single cell will incorporate multiple lentiviral particles. 4 days after adding the lentivirus, the culturing media was changed to blasticidin (10 μg/mL)-containing DMEM, high glucose, GlutaMAX™, HEPES media media (Gibco) to select for cells that incorporated Cas9-Blast. The blasticidin-containing DMEM media was replaced every 24 h until a control plate in parallel of the same quantity of non-Cas9-infected HeLa cells exhibited complete cell death. Cas9-BFP cells were clonally isolated using FACS.

#### Genome-wide deletion library production and tittering

All gRNA oligonucleotides were constructed on a microfluidic array, then lentivirus was generated using standard protocols. Briefly, all guides were pooled together at roughly the same concentration (10 sgRNAs per gene targeting ~21,000 human genes and ~10,000 safe-targeting sgRNAs), which were cloned into a lentiviral backbone. This pool was used to transfect low-passage HEK293T cells at 70–80% confluency in 150 mm dishes, from which the resulting supernatant contained all 25,000 sgRNAs, with each sgRNA represented ~1000 times.

#### Generating genome-wide knockout cell line

HeLa-Cas9 cells were cultured in DMEM, high glucose, GlutaMAX™, HEPES media (Gibco) containing 10% FBS (Omega) and 1% penicillin-streptomycin (P/S) (Gibco) in 150 mm plates. The viral media generated above—containing 1000x representation of each sgRNA—was used to infect the HeLa-Cas9 cells. The virus titering was performed such that 5–10% of cells were mCherry-positive, to reduce the chance that a single cell will incorporate multiple gRNAs. 24 h after infection, media was changed to DMEM media containing puromycin (1 μg/mL) to select for infected cells. The puromycin-containing media was replaced every 24 h until >90% of cells were mCherry-positive, and an uninfected control plate containing HeLa-Cas9 cells exhibited complete cell death when subjected to puromycin selection. The cells were grown for an additional five days to give Cas9 ample time to cut.

### Fixation and IF

After trypsinization, approximately 400 million cells of the genome-wide deletion cell line were fixed in 100% methanol for 10 min at −20°C for the case of immediate use. If fixing cells for long-term storage, the cells were moved directly into −80°C (these methanol-fixed cells can be thawed and utilized for immunostaining and FACS even after long-term storage). The number of cells to fix was chosen based on ensuring 1000x coverage of the whole genome (250,000 guide elements) while accounting for cells lost during fixation/staining/FACS. After placing in blocking solution (0.4% PBS-Triton containing 5% normal donkey serum and 0.5% BSA) for one hour, primary antibodies against ataxin-2 (1:100; Rabbit; ProteinTech 21776-1-AP) and house-keeping protein GAPDH (1:500; Mouse; Sigma-Aldrich G8795) or β-actin (1:100; Mouse; ThermoFisher Scientific MA1–744) were added to the sample and left overnight at 4°C on a shaker. After rinsing one time in PBS-Triton (0.4%), fluorescent secondary antibodies were added (1:500) for two hours. The cells were then rinsed in PBS, resuspended in PBS containing 2 mM EDTA, and taken directly to the FACS facility for sorting.

#### Fluorescence-activated cell sorting

To identify genetic modifiers of ataxin-2 protein levels, the cell suspension was sorted using a BD FACSAria II cell sorter with a 70 μm nozzle (BD Biosciences). Cell populations containing the lowest and highest 20% of ataxin-2 levels—relative to β-actin or GAPDH—were sorted, respectively. Each sorted population, as well as the unsorted (starting) population, were spun down at 600g for 20 min at room temperature before extracting genomic DNA.

#### Genomic DNA extraction, PCR amplification, and next-generation sequencing

For each sorted or unsorted population, genomic DNA was extracted immediately after pelleting using the Blood and Tissue DNeasy Maxi Kit (QIAGEN 51194). The DNA was isolated according to the manufacturer’s instructions, except for eluting with buffer EB, rather than buffer AE. The samples were prepared for deep sequencing using two sequential PCR reactions to 1) amplify sgRNA sequences and 2) barcode each sample (for later pooling all samples for sequencing and to then computationally deconvolute)—as described previously (Morgens et al., 2016)—using Agilent Herculase II Fusion DNA Polymerase Kit. The second PCR products were then run on a 2% TBE-agarose gel and gel purified using QIAquick Gel Extraction Kit (QIAGEN 28706). The concentrations of the purified second PCR products were measured using a Qubit. After pooling all samples and diluting them further according to NextSeq 500/550 High Output Kit v2.5 (Illumina FC-404–2001) instructions, deep sequencing was performed on an Illumina NextSeq 550 platform to determine library composition. Guide composition between the sorted top 20% and the unsorted (starting) populations were compared using Cas9 high Throughput maximum Likelihood Estimator (casTLE) (Morgens et al., 2016) to determine genes that, when knocked out, increased or decreased ataxin-2 protein levels. Briefly, enrichment of individual guides was calculated as median normalized log ratios of counts between the various conditions. Gene-level effects were then calculated from ten guides targeting each gene, and an effect size estimate was derived for each gene with an associated-likelihood ratio to describe the significance of the gene-level effects. By randomly permutating the targeting elements, the distribution of the log likelihood ratio was estimated and *p* values derived (Morgens et al., 2016).

### siRNA treatments

For knockdown experiments, HeLa cells were reverse transfected with SMARTPool ON-TARGETplus siRNAs (GE Dharmacon) targeting a control siRNA pool (D001810–10) or a gene of interest at a final concentration of 200 nM for 72 h in 12-well plates, after complexing with DharmafectI (GE Dharmacon) in Opti-MEM (Gibco) for 30 min siRNA reverse transfection experiments in SH-SY5Y cells were conducted similarly as for HeLa, except for performing knockdown for 96 h in two doses (a second dose given after 24 h with a full media change) and complexing with Lipofectamine RNAiMAX (Invitrogen) in Opti-MEM (Gibco) for 20 min prior to addition of cells. Cells were cultured in 24-well plates.

### Generating knockout cell lines using CRISPR-Cas9

To generate mosaic *ATXN2* KO HeLa-Cas9 cells, a sgRNA oligonucleotide targeting the first shared exon in *ATXN2* (sequence GATGGCATGGAGCCCGATCC) was cloned into a lentiviral backbone that contains mCherry and a puromycin resistance cassette. This construct was used to transfect low-passage HEK293T cells at 70–80% confluency in 100 mm plates. Third-generation packaging plasmids (pMDLg-pRRE, pRSV-Rev, pMD2.G(Addgene)) were used for lentivirus production. HeLa-Cas9 cells (cultured in DMEM, high glucose, GlutaMAX™, HEPES media containing 10% FBS and 1% penicillin-streptomycin in 100 mm plates) were then infected with the lentiviral media generated above, such that ~50% of cells were mCherry-positive. 24 h after infection, the media was changed to puromycin-containing media (1 μg/mL) to select for cells that received a sgRNA. The puromycin-containing media was replaced every 24 h until >90% of cells were mCherry-positive, and an uninfected control plate containing HeLa-Cas9 cells exhibited complete cell death upon subjection to puromycin selection. The cells were grown for an additional week to give Cas9 ample time to cut prior to use in flow cytometry. The same methods were used to target *ATP6V1A*, except using a sgRNA oligonucleotide targeting *ATP6V1A* (sequence GACACGTTTACTCCTCTG) and utilizing FACS after puromycin selection to achieve clonality.

### Western blots

Ice-cold RIPA buffer (Sigma-Aldrich R0278) containing protease inhibitor cocktail (Thermo Fisher 78429) and phosphatase inhibitor (Thermo Fisher 78426) was placed on cells for lysis. After 1–2 min, the lysates were moved to Protein LoBind tubes (Eppendorf 02243108), vortexed, and placed on ice. The lysates were vortexed two more times after 10 min intervals then pelleted at maximum speed on a table-top centrifuge for 15 minutes at 4°C. After moving the supernatant to new Protein LoBind tubes, protein concentrations were determined using bicinchoninic acid (Invitrogen 23225) assays. Samples were denatured at 70°C in LDS sample buffer (Invitrogen NP0008) containing 2.5% 2-mercaptoethanol (Sigma-Aldrich) for 10 min. Samples were run on 4–12% Bis–Tris gels (Thermo Fisher) using gel electrophoresis, then wet-transferred (Bio-Rad Mini Trans-Blot Electrophoretic Cell 170–3930) onto 0.45 μm nitrocellulose membranes (Bio-Rad 162–0115) at 100V for 90 min. Odyssey Blocking Buffer (LI-COR 927–40010) was applied to membranes for one hour then replaced with Odyssey Blocking Buffer containing antibodies against ataxin-2 (1:1000, ProteinTech 21776-1-AP) and β-actin (1:2000, Thermo Fisher Scientific MA1-744) and placed on a shaker overnight at room temperature. After rinsing three times in PBS-Tween (0.1%) for 10 min each, membranes were incubated in Odyssey Blocking Buffer containing HRP-conjugated anti-rabbit IgG (H + L) (1:2000, Life Technologies 31462) or anti-mouse IgG (H + L) (1:2000, Fisher 62–6520) secondary antibodies for one hour. After rinsing the blots three additional times in PBS-Tween (0.1%), the membranes were developed using ECL Prime kit (Invitrogen) and imaged using ChemiDoc XRS + System and Image Lab software (Bio-Rad Laboratories).

### Quantitative reverse transcription PCR (RT-qPCRs) and RNA quantification

After reverse transfection with siRNAs in 12-well plates as described in the ‘Cell culture and siRNA transfection’ section, the PureLink® RNA Mini Kit was used to isolate RNA with DNase digestion (Thermo Fisher Scientific 12183025). To convert RNA to cDNA, we used the Applied Biosystems High-Capacity cDNA Reverse Transcription kit (Thermo Fisher Scientific 4368813). Each sample had biological triplicates, and technical quadruplicates for each of the replicates. qPCR was performed using TaqMan™ Universal Master Mix II (Thermo Fisher Scientific4440040), using 1 μL of 20X TaqMan gene-specific expression assay to the reaction and our probes of interest (Thermo Fisher Scientific; human *ATXN2*: Hs00268077_m1, human *ACTB*: Hs01060665_g1). The Delta-Delta Ct method was run on the thermocycler and visualized on Thermo Fisher Connect™, from which relative expression values were averaged across all biological/technical replicates per condition.

### RNA-sequencing and RNA-sequencing analysis

To determine whether there are broad transcriptional changes after knocking down a v-ATPase subunit, we performed RNA-seq after HeLa cells were treated with NT or *ATP6V1A* siRNAs for 72 h, as described in the ‘siRNA treatment’ section. Briefly, we isolated RNA using the PureLink® RNA Mini Kit with DNase digestion (Thermo Fisher Scientific 12183025), then determined RNA quantity and purity by optical density measurements of OD260/280 and OD230/260 using a NanoDrop spectrophotometer. We assessed structural integrity of the total RNA using a 2200 TapeStation Instrument with RNA ScreenTapes (Agilent Technologies), then prepared mRNA libraries using SureSelect Strand-Specific RNA Library Preparation kit for Illumina (G9691B) on an Agilent Bravo Automated Liquid Handling Platform, following the manufacturer’s protocol. Libraries were sequenced on an Illumina Nova-Seq 6000 machine. Once the data was retrieved, alignment of RNA-sequencing reads to the human hg38 transcriptome was performed using STAR v2.7.3a (Dobin et al., 2013) following ENCODE standard options, read counts were generated using RSEM v1.3.1, and differential expression analysis was performed in R v4.0.2 using the DESeq2 package v1.28.1 (Love et al., 2014) (detailed pipeline v2.1.2 and options available on https://github.com/emc2cube/Bioinformatics/). All data is available under Gene Expression Omnibus accession no. GSE189417.

#### *In vitro* drug treatments

##### Mouse primary cortical neurons

Mouse primary cortical neurons were plated according to the ‘Mouse primary cortical neurons’ section under ‘experimental model and subject details.’ After 4 days *in vitro* (DIV), a full media change was performed containing various concentrations of Etidronate (ranging from 1 nM to 100 μM) or water (control), Alendronate (ranging from 1 nM to 10 μM) or water (control), or Thonzonium (ranging from 1 nM to 10 μM) or DMSO (control) prior to further processing steps. For the stress granule assay, we added 0.5 mM sodium arsenite to the cells for the final hour prior to fixing the cells using 4% paraformaldehyde (next steps for stress granule visualization outlined in ‘immunocytochemistry, microscopy, and image quantification’ section).

##### iPSC-derived neurons

Human iPSC-derived neurons were cultured following the procedure described in the ‘neuron differentiation from iPSCs’ section under ‘experimental model and subject details.’ 6–7 days after Ngn2 induction, the cells were treated with various doses of Etidronate (dissolved in media) or water (control, all to equal volumes) and lysed 24 h later for protein collection.

##### SH-SY5Y cells

SH-SY5Y cells were seeded at a density of 5 × 10^5^ cells per well in 24-well plates. One day after plating, the cells were treated with various doses of Etidronate, Alendronate, or Thonzonium (dissolved in media, all to equal volumes) and lysed 24 h later for protein collection.

##### Immunocytochemistry, microscopy, and image quantification

WT or *ATP6V1A* Cas9-edited HeLa cells were grown on poly-L-lysine-coated glass coverslips [0.1% (wt/vol)] in 24-well plates (four wells per condition), then fixed with 4% paraformaldehyde for 30 min. Next, the cells were rinsed 3 times with PBS then blocked with 1% BSA containing 0.4% Triton X-100 for one hour. After overnight primary antibody incubation (1:1000 ataxin-2, ProteinTech 21776-1-AP; 1:1000 β-actin, Thermo Fisher Scientific MA1-744), cells were rinsed 3 times with PBS prior to incubating with fluorescent secondary antibodies (1:500) for one hour. After rinsing with PBS 3 times, coverslips were mounted using Prolong Diamond Antifade mount containing DAPI (Thermo Fisher Scientific). All steps were carried out at room temperature. Images were acquired using a Zeiss LSM 710 confocal microscope (three fields-of-view per coverslip, four coverslips per condition). Images were processed and analyzed using ImageJ (version 2.1.0). Quantification of fluorescence intensities was conducted on >270 cells per condition.

To determine whether Etidronate treatment led to changes in TDP-43 protein levels and localization via immunocytochemistry, we followed the same protocol as above, except that we plated primary neurons from mouse embryos (E16.5) in 24-well plates on glass coverslips at 350,000 cells/well density. In brief, after treating these neurons with H_2_O or 10 μM Etidronate for 24 h (four wells per condition), we immunostained the cells using antibodies against TDP-43 (1:1000, ProteinTech 10782-2-AP) and MAP2 (1:1000, Synaptic Systems 188 004), then mounted coverslips onto slides using Prolong Diamond Antifade mount containing DAPI (Thermo Fisher Scientific). We also immunostained cells with the same treatment conditions for ataxin-2 (1:1000, Novus NBP1-90063) and MAP2. For the stress granule assay, we added 0.5 mM sodium arsenite to the cells for the final hour prior to the 4% paraformaldehyde fixation step and utilized antibodies against PABP (1:1000, Abcam ab21060) and MAP2, followed by Prolong Diamond Antifade mount containing DAPI (Thermo Fisher Scientific). Images were acquired using a Zeiss LSM 710 confocal microscope (20~30 images taken per condition over four coverslips) then processed and analyzed using ImageJ (version 2.1.0). We analyzed 250 or more cells per condition. For stress granule quantification, the numbers of cells containing PABP-positive stress granules—as well as total number of cells—were counted for each image. We calculated the proportion of SG-containing cells/total cells for each image, which would each count as a single data point. All image quantifications were conducted by a person blind to the treatment condition.

#### *In vivo* drug treatments

3–4-month-old WT C57BL/6J mice were given normal (control group) or Etidronate-infused water (~2 μg/mL) and MediGel® Sucralose (ClearH_2_O) (~20 mg/kg/day concentration) (treatment group) for voluntary consumption, with 16 animals in the control group (8 males, 8 females) and 14 animals in the treatment group (7 males, 7 females). After one week, the animals perfused with PBS before dissecting out their brains for flash-freezing. Olfactory bulbs were removed, the hemispheres were separated, and each hemisphere was divided into cortex and cerebellum chunks. Left cortices were then Dounce homogenized and treated for protein extraction in a conventional manner, as described above in the ‘Immunoblots’ section.

### QUANTIFICATION AND STATISTICAL ANALYSIS

Analyses were performed using RStudio (version 1.3.959) or Prism 9.0 (GraphPad), and graphs were generated using Prism 9.0. Data represent mean ± SD. Specific tests (e.g., unpaired t-test, one-way ANOVA, two-way ANOVA, post-hoc tests) and significance are indicated in figure legends.

## Supplementary Material

1

2

## Figures and Tables

**KEY RESOURCES TABLE:** 

REAGENT or RESOURCE	SOURCE	IDENTIFIER

Antibodies		

Ataxin-2	ProteinTech	Cat# 21776-1-AP; RRID: AB_10858483
Ataxin-2	Novus	Cat# NBP1-90063; RRID: AB_11028587
GAPDH	Sigma-Aldrich	Cat# G8795; RRID: AB_1078991
Actin (mAbGEa)	ThermoFisher Scientific	Cat# MA1-744; RRID: AB_2223496
TDP-43	ProteinTech	Cat# 10782-2-AP; RRID: AB_615042
MAP2	Synaptic Systems	Cat# 188 004; RRID: AB_2138181
PABP	Abcam	Cat# ab21060; RRID: AB_777008
ATP6V1A	Abcam	Cat# ab137574; RRID: AB_2722516
Ataxin-1	Cell Signaling Technology	Cat# 2177S; RRID: AB_2061047
Huntingtin	Abcam	Cat# ab109115; RRID: AB_10863082
Goat anti-Rabbit IgG (H + L) cross-adsorbed secondary antibody, HRP	Life Technologies	Cat# 31462
Goat anti-Mouse IgG (H + L) secondary antibody, HRP	Fisher Scientific	Cat# 6206520
Alexa Fluor 488 Donkey anti-Mouse IgG (H + L)	Thermo Fisher Scientific	Cat# A21202
Alexa Fluor 647 Donkey anti-Rabbit IgG (H + L)	Thermo Fisher Scientific	Cat# A31573

Chemicals, peptides, and recombinant proteins		

DharmaFECT 1 Transfection Reagent	Horizon Discovery	Cat# T-2001-03
Lipofectamine RNAiMAX	Invitrogen	Cat# 13778-075
Molecular Probes Prolong Gold Antifade Mountant	Thermo Fisher Scientific	Cat# P36931
Etidronate	MedChem Express	Cat# HY-B0302
Alendronate	MedChem Express	Cat# HY-B0631
Thonzonium	ApexBio	Cat# B4962
Sodium arsenite	Sigma-Aldrich	Cat# S7400

Critical commercial assays		

Nextseq 500/550 High Output v2 kit (75 cycles)	Illumina	Cat# FC-404-2005
SureSelect Strand-Specific RNA Library Preparation kit	Illumina	Cat# G9691-90010

Deposited data		

Raw and analyzed data	This paper	GEO: GSE189417

Experimental models: Cell lines		

293T	ATCC	CRL-3216
HeLa	ATCC	CRM-CCL-2
SH-SY5Y	ATCC	CRL-2266
NGN2-iPSCs	Ma et al. (2022)	N/A

Experimental models: Organisms/strains		

Mouse: C57BL/6J	The Jackson Laboratory	Strain# 000664; RRID: IMSR_JAX:000664

Oligonucleotides		

ON-TARGETplus Non-targeting Pool	Horizon Technology	D-001810-10-20
ON-TARGETplus Human ALG1 siRNA	Horizon Technology	L-027147-01-0005
ON-TARGETplus Human ATP6V1A siRNA	Horizon Technology	L-017590-01-0005
ON-TARGETplus Human ATP6V1B2 siRNA	Horizon Technology	L-011589-01-0005
ON-TARGETplus Human ATP6V1C1 siRNA	Horizon Technology	L-013139-01-0005
ON-TARGETplus Human ATP6V1D siRNA	Horizon Technology	L-013221-00-0005
ON-TARGETplus Human ATP6V1H siRNA	Horizon Technology	L-010930-00-0005
ON-TARGETplus Human ATXN2 siRNA	Horizon Technology	L-011772-00-0010
ON-TARGETplus Human ATXN7L3 siRNA	Horizon Technology	L-023237-01-0005
ON-TARGETplus Human CFAP20 siRNA	Horizon Technology	L-020289-02-0005
ON-TARGETplus Human CLASRP siRNA	Horizon Technology	L-019710-02-0005
ON-TARGETplus Human CMTR2 siRNA	Horizon Technology	L-018783-01-0005
ON-TARGETplus Human DPAGT1 siRNA	Horizon Technology	L-011255-01-0005
ON-TARGETplus Human ENY2 siRNA	Horizon Technology	L-018808-01-0005
ON-TARGETplus Human LSM12 siRNA	Horizon Technology	L-015641-01-0005
ON-TARGETplus Human LUC7L3 siRNA	Horizon Technology	L-015383-00-0005
ON-TARGETplus Human PAXBP1 siRNA	Horizon Technology	L-012984-02-0005
ON-TARGETplus Human PNISR siRNA	Horizon Technology	L-015028-02-0005
ON-TARGETplus Human RFT1 siRNA	Horizon Technology	L-018174-02-0005
ON-TARGETplus Human SNRPA siRNA	Horizon Technology	L-019435-02-0005
ON-TARGETplus Human TSC1 siRNA	Horizon Technology	L-003028-00-0005
ON-TARGETplus Human TSC2 siRNA	Horizon Technology	L-003029-00-0005
ON-TARGETplus Human UBA3 siRNA	Horizon Technology	L-005249-00-0005
ON-TARGETplus Human USP22 siRNA	Horizon Technology	L-006072-03-0005
ON-TARGETplus Human VHL siRNA	Horizon Technology	L-003936-00-0005

Recombinant DNA		

pMDLg-pRRE	Addgene	Cat# 12251
pRSV-Rev	Addgene	Cat# 12253
pMD2.G	Addgene	Cat# 12259

Software and algorithms		

ImageJ	National Institute of Health	https://imagej.nih.gov/ij/; RRID: SCR_003073
CRISPR screen analysis	Kramer et al. (2018)	https://github.com/emc2cube/Bioinformatics/blob/master/sh_CRISPR.sh
RNA-seq analysis	Kramer et al., 2018	https://github.com/emc2cube/Bioinformatics/blob/master/sh_RNAseq.sh

Other		

MediGel Sucralose	ClearH2O	Cat# 74-02-5022

## Highlights

- Establish a FACS-based CRISPR-Cas9 genome-wide screening method using fixed cells
- Identify modifiers of levels of the ALS and SCA2 protein ataxin-2
- Genetic or pharmacologic inhibition of v-ATPase leads to decreased ataxin-2 *in vitro*
- Oral intake of small molecule v-ATPase inhibitor by adult mice lowers ataxin-2 in brain

## References

[R1] Altman RD, Johnston CC, Khairi MR, Wellman H, Serafini AN, and Sankey RR (1973). Influence of disodium etidronate on clinical and laboratory manifestations of Paget’s disease of bone (osteitis deformans). N. Engl. J. Med. 289, 1379–1384. 10.1056/NEJM197312272892601. 4201876

[R2] Armakola M, Hart MP, and Gitler AD (2011). TDP-43 toxicity in yeast. Methods 53, 238–245. 10.1016/j.ymeth.2010.11.006. 21115123 PMC3073690

[R3] Arnold ES, Ling S-C, Huelga SC, Lagier-Tourenne C, Polymenidou M, Ditsworth D, Kordasiewicz HB, McAlonis-Downes M, Platoshyn O, Parone PA, (2013). ALS-linked TDP-43 mutations produce aberrant RNA splicing and adult-onset motor neuron disease without aggregation or loss of nuclear TDP-43. Proc. Natl. Acad. Sci. USA 110, E736–E745. 10.1073/pnas.1222809110. 23382207 PMC3581922

[R4] Becker LA, Huang B, Bieri G, Ma R, Knowles DA, Jafar-Nejad P, Messing J, Kim HJ, Soriano A, Auburger G, (2017). Therapeutic reduction of ataxin-2 extends lifespan and reduces pathology in TDP-43 mice. Nature 544, 367–371. 10.1038/nature22038. 28405022 PMC5642042

[R5] Bennett CF, Kordasiewicz HB, and Cleveland DW (2021). Antisense drugs make sense for neurological diseases. Annu. Rev. Pharmacol. Toxicol. 61, 831–852. 10.1146/annurev-pharmtox-010919-023738. 33035446 PMC8682074

[R6] Chan C-Y, Prudom C, Raines SM, Charkhzarrin S, Melman SD, De Haro LP, Allen C, Lee SA, Sklar LA, and Parra KJ (2012). Inhibitors of V-ATPase proton transport reveal uncoupling functions of tether linking cytosolic and membrane domains of V0 subunit a (Vph1p). J. Biol. Chem. 287, 10236–10250. 10.1074/jbc.M111.321133. 22215674 PMC3323027

[R7] David P, Nguyen H, Barbier A, and Baron R (1996). The bisphosphonate tiludronate is a potent inhibitor of the osteoclast vacuolar H(+)-ATPase. J. Bone Miner. Res. 11, 1498–1507. 10.1002/jbmr.5650111017. 8889850

[R8] de Boer EMJ, Orie VK, Williams T, Baker MR, De Oliveira HM, Polvikoski T, Silsby M, Menon P, van den Bos M, Halliday GM, (2020). TDP-43 proteinopathies: a new wave of neurodegenerative diseases. J. Neurol. Neurosurg. Psychiatry 92, 86–95. 10.1136/jnnp-2020-322983. PMC7803890 33177049

[R9] Dobin A, Davis CA, Schlesinger F, Drenkow J, Zaleski C, Jha S, Batut P, Chaisson M, and Gingeras TR (2013). STAR: ultrafast universal RNA-seq aligner. Bioinformatics 29, 15–21. 10.1093/bioinformatics/bts635. 23104886 PMC3530905

[R10] Drake MT, Clarke BL, and Khosla S (2008). Bisphosphonates: mechanism of action and role in clinical practice. Mayo Clin. Proc. 83, 1032–1045. 10.4065/83.9.1032. 18775204 PMC2667901

[R11] Elden AC, Kim H-J, Hart MP, Chen-Plotkin AS, Johnson BS, Fang X, Armakola M, Geser F, Greene R, Lu MM, (2010). Ataxin-2 intermediate-length polyglutamine expansions are associated with increased risk for ALS. Nature 466, 1069–1075. 10.1038/nature09320. 20740007 PMC2965417

[R12] Forman MS, Trojanowski JQ, and Lee VM-Y (2004). Neurodegenerative diseases: a decade of discoveries paves the way for therapeutic breakthroughs. Nat. Med. 10, 1055–1063. 10.1038/nm1113. 15459709

[R13] Gitcho MA, Strider J, Carter D, Taylor-Reinwald L, Forman MS, Goate AM, and Cairns NJ (2009). VCP mutations causing frontotemporal lobar degeneration disrupt localization of TDP-43 and induce cell death. J. Biol. Chem. 284, 12384–12398. 10.1074/jbc.M900992200. 19237541 PMC2673306

[R14] Hart MP, and Gitler AD (2012). ALS-associated ataxin 2 polyQ expansions enhance stress-induced caspase 3 activation and increase TDP-43 pathological modifications. J. Neurosci. 32, 9133–9142. 10.1523/JNEUROSCI.0996-12.2012. 22764223 PMC3418890

[R15] Imbert G, Saudou F, Yvert G, Devys D, Trottier Y, Garnier JM, Weber C, Mandel JL, Cancel G, Abbas N, (1996). Cloning of the gene for spinocerebellar ataxia 2 reveals a locus with high sensitivity to expanded CAG/glutamine repeats. Nat. Genet. 14, 285–291. 10.1038/ng1196-285. 8896557

[R16] Kim G, Gautier O, Tassoni-Tsuchida E, Ma XR, and Gitler AD (2020). ALS genetics: gains, losses, and implications for future therapies. Neuron 108, 822–842. 10.1016/j.neuron.2020.08.022. 32931756 PMC7736125

[R17] Kim H-J, Raphael AR, LaDow ES, McGurk L, Weber RA, Trojanowski JQ, Lee VM-Y, Finkbeiner S, Gitler AD, and Bonini NM (2014). Therapeutic modulation of eIF2α phosphorylation rescues TDP-43 toxicity in amyotrophic lateral sclerosis disease models. Nat. Genet. 46, 152–160. 10.1038/ng.2853. 24336168 PMC3934366

[R18] Kramer NJ, Haney MS, Morgens DW, Jovičić A, Couthouis J, Li A, Ousey J, Ma R, Bieri G, Tsui CK, (2018). CRISPR–Cas9 screens in human cells and primary neurons identify modifiers of C9ORF72 dipeptide-repeat-protein toxicity. Nat. Genet. 50, 603–612. 10.1038/s41588-018-0070-7. 29507424 PMC5893388

[R19] Li YR, King OD, Shorter J, and Gitler AD (2013). Stress granules as crucibles of ALS pathogenesis. J. Cell Biol. 201, 361–372. 10.1083/jcb.201302044. 23629963 PMC3639398

[R20] Love MI, Huber W, and Anders S (2014). Moderated estimation of fold change and dispersion for RNA-seq data with DESeq2. Genome Biol. 15, 550. 10.1186/s13059-014-0550-8. 25516281 PMC4302049

[R21] Lu B, Al-Ramahi I, Valencia A, Wang Q, Berenshteyn F, Yang H, Gallego-Flores T, Ichcho S, Lacoste A, Hild M, (2013). Identification of NUB1 as a suppressor of mutant Huntington toxicity via enhanced protein clearance. Nat. Neurosci. 16, 562–570. 10.1038/nn.3367. 23525043

[R22] Ma XR, Prudencio M, Koike Y, Vatsavayai SC, Kim G, Harbinski F, Briner A, Rodriguez CM, Guo C, Akiyama T, (2022). TDP-43 represses cryptic exon inclusion in the FTD-ALS gene UNC13A. Nature 603, 124–130. 35197626 10.1038/s41586-022-04424-7 PMC8891019

[R23] Maxson ME, and Grinstein S (2014). The vacuolar-type H^+^-ATPase at a glance - more than a proton pump. J. Cell Sci. 127, 4987–4993. 10.1242/jcs.158550. 25453113

[R24] Morgens DW, Deans RM, Li A, and Bassik MC (2016). Systematic comparison of CRISPR/Cas9 and RNAi screens for essential genes. Nat. Biotechnol. 34, 634–636. 10.1038/nbt.3567. 27159373 PMC4900911

[R25] Morgens DW, Wainberg M, Boyle EA, Ursu O, Araya CL, Tsui CK, Haney MS, Hess GT, Han K, Jeng EE, (2017). Genome-scale measurement of off-target activity using Cas9 toxicity in high-throughput screens. Nat. Commun. 8, 15178. 10.1038/ncomms15178. 28474669 PMC5424143

[R26] Neumann M, Mackenzie IR, Cairns NJ, Boyer PJ, Markesbery WR, Smith CD, Taylor JP, Kretzschmar HA, Kimonis VE, and Forman MS (2007). TDP-43 in the ubiquitin pathology of frontotemporal dementia with VCP gene mutations. J. Neuropathol. Exp. Neurol. 66, 152–157. 10.1097/nen.0b013e31803020b9. 17279000

[R27] Neumann M, Sampathu DM, Kwong LK, Truax AC, Micsenyi MC, Chou TT, Bruce J, Schuck T, Grossman M, Clark CM, (2006). Ubiquitinated TDP-43 in frontotemporal lobar degeneration and amyotrophic lateral sclerosis. Science 314, 130–133. 10.1126/science.1134108. 17023659

[R28] Park J, Al-Ramahi I, Tan Q, Mollema N, Diaz-Garcia JR, Gallego-Flores T, Lu H-C, Lagalwar S, Duvick L, Kang H, (2013). RAS-MAPK-MSK1 pathway modulates ataxin 1 protein levels and toxicity in SCA1. Nature 498, 325–331. 10.1038/nature12204. 23719381 PMC4020154

[R29] Pulst SM, Nechiporuk A, Nechiporuk T, Gispert S, Chen XN, LopesCendes I, Pearlman S, Starkman S, Orozco-Diaz G, Lunkes A, (1996). Moderate expansion of a normally biallelic trinucleotide repeat in spinocerebellar ataxia type 2. Nat. Genet. 14, 269–276. 10.1038/ng1196-269. 8896555

[R30] Rodriguez CM, Bechek SC, Jones GL, Nakayama L, Akiyama T, Kim G, Solow-Cordero DE, Strittmatter SM, and Gitler AD (2022). Targeting RTN4/NoGo-Receptor reduces levels of ALS protein ataxin-2. Cell Rep. 41. 10.1101/2021.12.20.473562. PMC9664481 36288715

[R31] Rossi A, Kontarakis Z, Gerri C, Nolte H, Hölper S, Krüger M, and Stainier DYR (2015). Genetic compensation induced by deleterious mutations but not gene knockdowns. Nature 524, 230–233. 10.1038/nature14580. 26168398

[R32] Rousseaux MWC, Vázquez-Vélez GE, Al-Ramahi I, Jeong H-H, Bajić A, Revelli J-P, Ye H, Phan ET, Deger JM, Perez AM, (2018). A druggable genome screen identifies modifiers of α-synuclein levels via a tiered cross-species validation approach. J. Neurosci. 38, 9286–9301. 10.1523/JNEUROSCI.0254-18.2018. 30249792 PMC6199406

[R33] Sanpei K, Takano H, Igarashi S, Sato T, Oyake M, Sasaki H, Wakisaka A, Tashiro K, Ishida Y, Ikeuchi T, (1996). Identification of the spinocerebellar ataxia type 2 gene using a direct identification of repeat expansion and cloning technique, DIRECT. Nat. Genet. 14, 277–284. 10.1038/ng1196-277. 8896556

[R34] Scoles DR, Meera P, Schneider MD, Paul S, Dansithong W, Figueroa KP, Hung G, Rigo F, Bennett CF, Otis TS, and Pulst SM (2017). Antisense oligonucleotide therapy for spinocerebellar ataxia type 2. Nature 544, 362–366. 10.1038/nature22044. 28405024 PMC6625650

[R35] Scoles DR, and Pulst SM (2018). Spinocerebellar ataxia type 2. Adv. Exp. Med. Biol. 1049, 175–195. 10.1007/978-3-319-71779-1_8. 29427103

[R36] Watts GDJ, Wymer J, Kovach MJ, Mehta SG, Mumm S, Darvish D, Pestronk A, Whyte MP, and Kimonis VE (2004). Inclusion body myopathy associated with Paget disease of bone and frontotemporal dementia is caused by mutant valosin-containing protein. Nat. Genet. 36, 377–381. 10.1038/ng1332. 15034582

[R37] Wils H, Kleinberger G, Janssens J, Pereson S, Joris G, Cuijt I, Smits V, Ceuterick-de Groote C, Van Broeckhoven C, and Kumar-Singh S (2010). TDP-43 transgenic mice develop spastic paralysis and neuronal inclusions characteristic of ALS and frontotemporal lobar degeneration. Proc. Natl. Acad. Sci. USA 107, 3858–3863. 10.1073/pnas.0912417107. 20133711 PMC2840518

[R38] Yin J-A, Frick L, Scheidmann MC, Trevisan C, Dhingra A, Spinelli A, Wu Y, Yao L, Vena DL, De Cecco E, (2022). Robust and versatile arrayed libraries for human genome-wide CRISPR activation, deletion and silencing. Preprint at bioRxiv. 10.1101/2022.05.25.493370. PMC11754104 39633028

[R39] Zhang K, Daigle JG, Cunningham KM, Coyne AN, Ruan K, Grima JC, Bowen KE, Wadhwa H, Yang P, Rigo F, (2018). Stress granule assembly disrupts nucleocytoplasmic transport. Cell 173, 958–971.e17. 10.1016/j.cell.2018.03.025. 29628143 PMC6083872

