# Supplementary material for: Genome-wide CRISPR screen reveals v-ATPase as a drug target to lower levels of ALS protein ataxin-2
Source: Cell Rep. Author manuscript; Available in PMC 2022 Nov 15. (PMC9664452; doi:10.1016/j.celrep.2022.111508)
Supplement: 1 [file NIHMS1845179-supplement-1.pdf]

Cell Reports, Volume *41*

Supplemental information

Genome-wide CRISPR screen reveals v-ATPase as a drug target to lower levels of ALS protein ataxin-2

Garam Kim, Lisa Nakayama, Jacob A. Blum, Tetsuya Akiyama, Steven Boeynaems, Meenakshi Chakraborty, Julien Couthouis, Eduardo Tassoni-Tsuchida, Caitlin M. Rodriguez, Michael C. Bassik, and Aaron D. Gitler

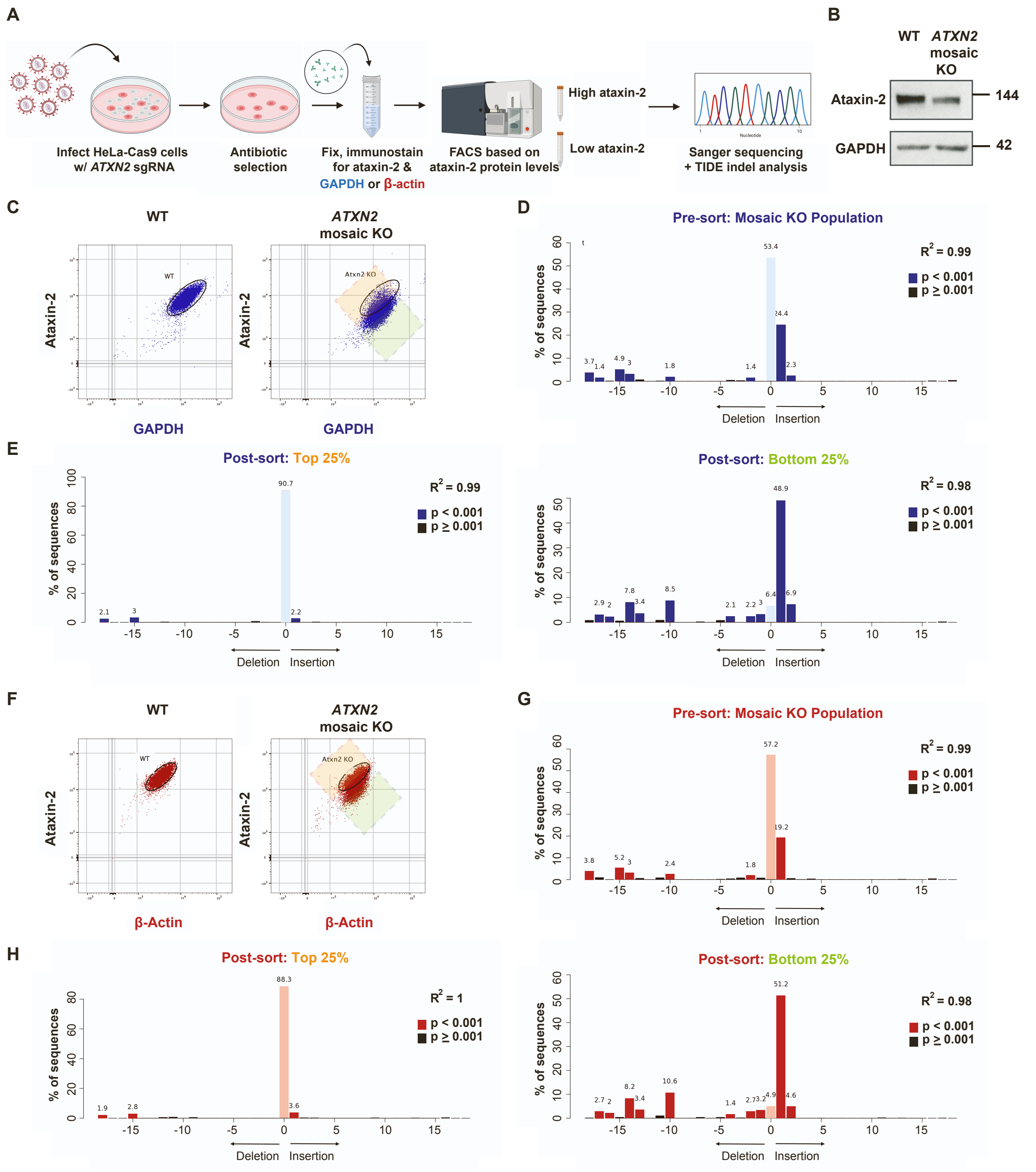

Figure S1: Calibration steps prior to conducting genome-wide screens, related to Figure 1. **(A)** Overview of screen optimization strategy. Briefly, HeLa cells expressing Cas9 were infected with a lentiviral sgRNA targeting *ATXN2*, then treated with puromycin to select for cells that received a guide. Cells were kept pooled to retain a mosaic population. These cells were then fixed in methanol, immunostained, and sorted using FACS for the top and bottom 25% of ataxin-2 expressors relative to a control protein (GAPDH or β-actin). The sorted and unsorted populations

were Sanger sequenced and analyzed for insertions or deletions (indels) at the *ATXN2* locus. **(B)** Immunoblot of the WT and *ATXN2* mosaic KO populations, as generated in panel a. **(C)** Gating strategy for FACS. Left, FACS plot for WT population around which a gate is drawn; Right, mosaic population relative to the WT population. The green and orange gates represent the bottom and top 25% of *ATXN2* expressors relative to GAPDH, respectively. **(D)** Indel (insertion and deletion) analysis of the unsorted mosaic population (i.e. FACS plot in panel c, Right) when Sanger sequenced at the *ATXN2* locus, showing a mixture of cells containing various indels. **(E)** Indel analysis at *ATXN2* locus for the sorted populations. Left, overrepresentation of *ATXN2* WT cells in the top 25% population (orange gate in panel c); Right, underrepresentation of WT cells / mostly various indels in the bottom 25% sorted population (green gate in panel c). **(F)** Same as in panel C, except using β-actin as control. **(G)** Same as in panel D, except using β-actin as control. **(H)** Same as in panel E, except using β-actin as control.

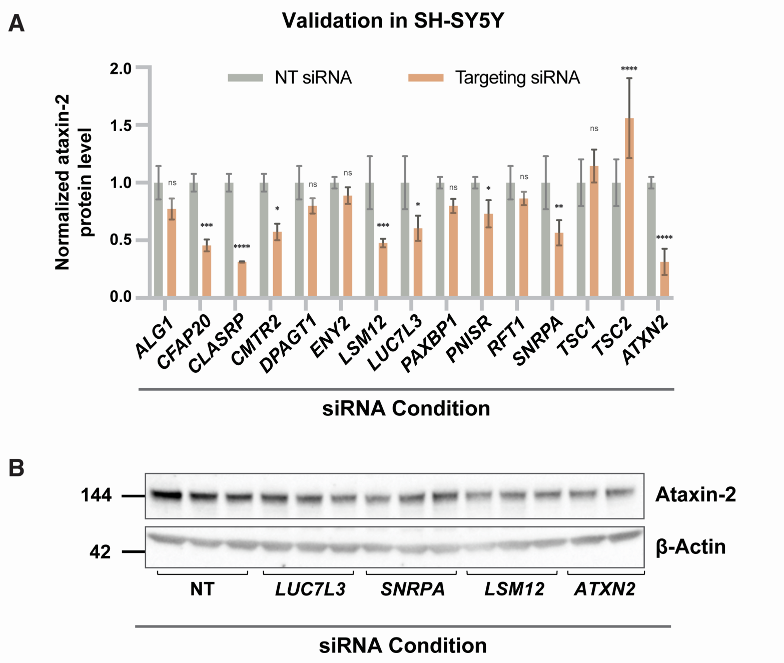

Figure S2: Validation of screen results in neuroblastoma cell line SH-SY5Y, related to Figure 1. (A) Validation of numerous top hit genes in SH-SY5Y cells using siRNA transfections and immunoblot analyses as in Fig. 1 C and D. Quantifications are normalized to the NT siRNA condition (mean ± SD; analyzed using 2-way ANOVA; ****: $p<0.0001$, ***: $p<0.001$, **: $p<0.01$, *: $p<0.05$, ns: not significant). **(B)** Representative immunoblot of ataxin-2 and β-actin protein levels upon application of NT, *LUC7L3, SNRPA, LSM12,* and *ATXN2* siRNAs to SH-SY5Y cells.

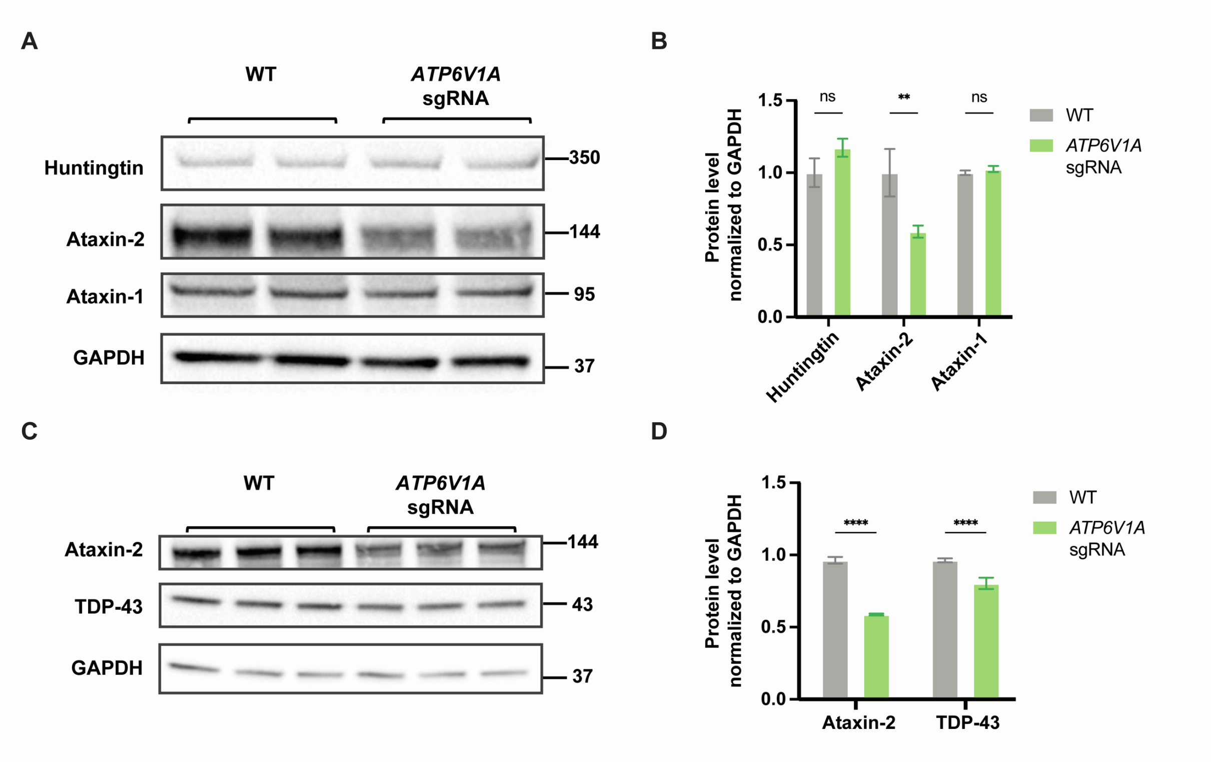

Figure S3: Other polyQ protein levels are unaltered and TDP-43 is slightly decreased in *ATP6V1A* Cas9-edited cells, related to Figure 3. (A) Immunoblot on lysates from *ATP6V1A* Cas9-edited HeLa cells probed for huntingtin, ataxin-2, ataxin-1, and GAPDH. **(B)** Quantification of immunoblot in panel (A), normalized to GAPDH (loading control) levels and to the WT cell line (mean ± SD). **(C)** Immunoblot on same lysates as (A) reveals a moderate decrease in TDP-43 protein levels, as quantified in **(D)** (mean ± SD)**.** For both (B) and (D), the data was analyzed using 2-way ANOVA with post-hoc Šídák's multiple comparisons tests (****: $p<0.001$, **: $p<0.01$, ns: not significant).

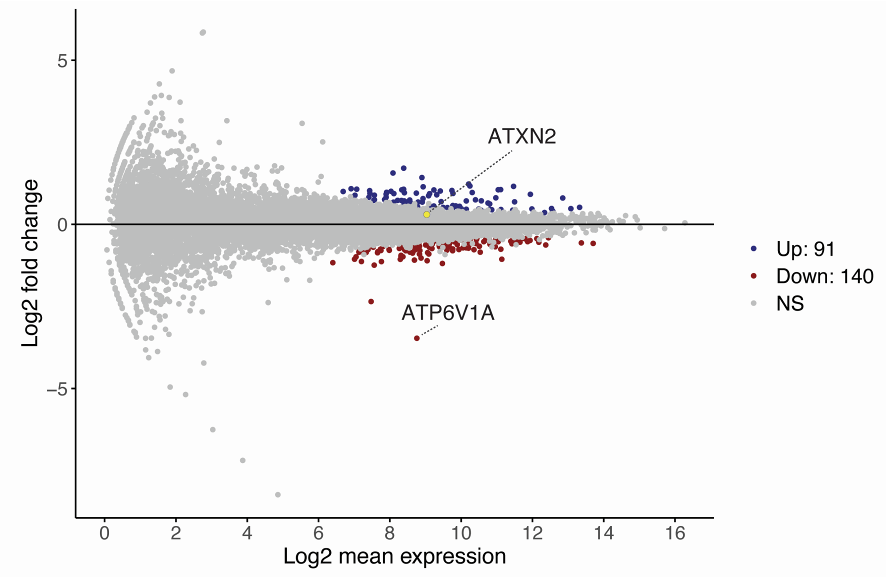

Figure S4: MA plot 72 hours after treatment with NT vs. *ATP6V1A* siRNAs in HeLa cells, related to Figure 3. To determine whether there are broad transcriptional changes after knocking down a v-ATPase subunit, we performed RNA-seq after HeLa cells were treated with NT or *ATP6V1A* siRNAs. Few noteworthy transcriptional changes are seen (apart from *ATP6V1A* itself) upon knockdown of *ATP6V1A* (FDR<0.01). Importantly, *ATXN2* mRNA levels were not altered by *ATP6V1A* knockdown (yellow circle).

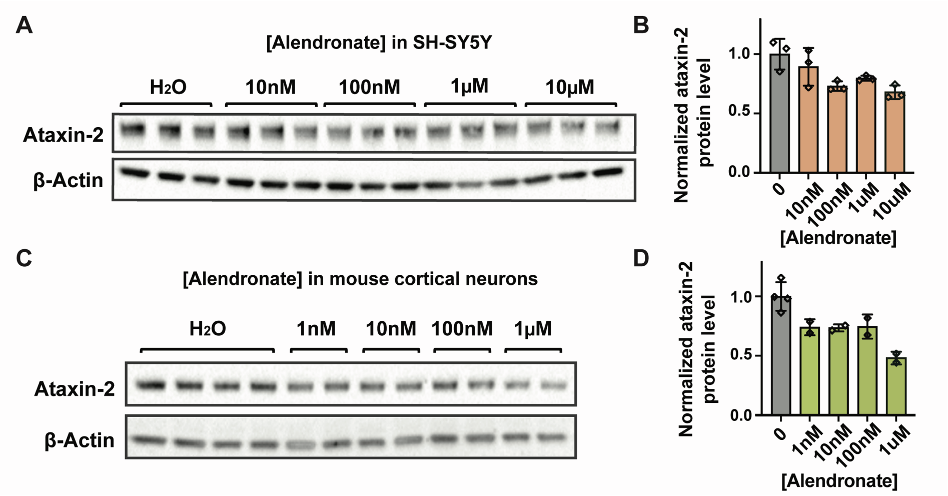

Figure S5: Treating human SH-SY5Y cells or mouse cortical neurons with another bisphosphonate leads to decreased ataxin-2 protein levels, related to Figure 4. **(A)** Immunoblot on lysates from human SH-SY5Y cells with various doses of Alendronate. **(B)** Quantification of the immunoblot in (B) reveals a dose-dependent effect of Alendronate on ataxin-2 protein levels (mean ± SD, normalized to control condition). **(C)** Immunoblot on lysates from mouse primary neurons treated with various doses of Alendronate. **(D)** Quantification of the dose-dependent effect of Alendronate on ataxin-2 (mean ± SD, normalized to control condition).

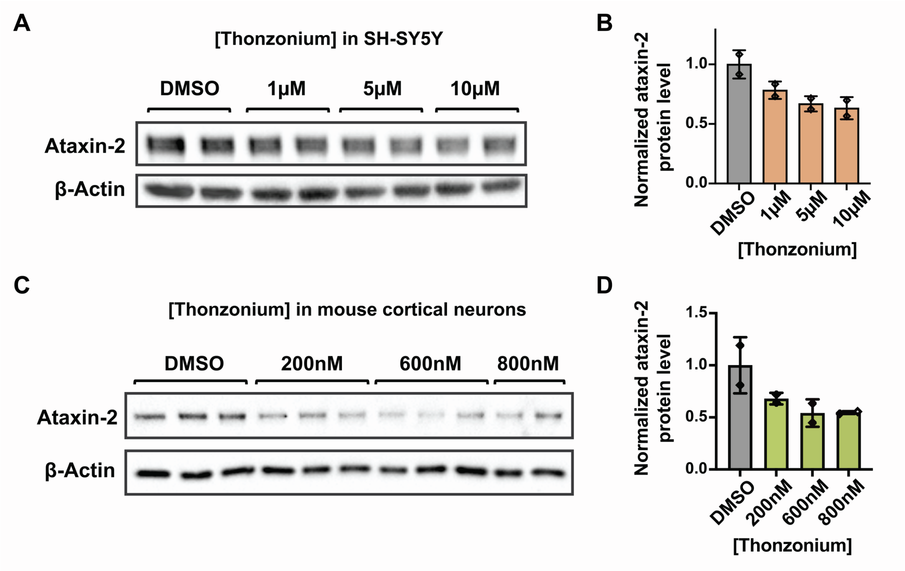

Figure S6: Treating human SH-SY5Y or mouse cortical neurons with Thonzonium, another small molecule drug, leads to decreased ataxin-2 protein levels, related to Figure 4. (A) Immunoblot on lysates from human SH-SY5Y cells with various doses of Thonzonium. **(B)** Quantification of the immunoblot in (A) reveals a dose-dependent effect of Thonzonium on ataxin-2 protein levels (mean ± SD, normalized to control condition). **(C)** Immunoblot on lysates from mouse primary neurons treated with various doses of Thonzonium. **(D)** Quantification of the dose-dependent effect of Thonzonium on ataxin-2 (mean ± SD, normalized to control condition).

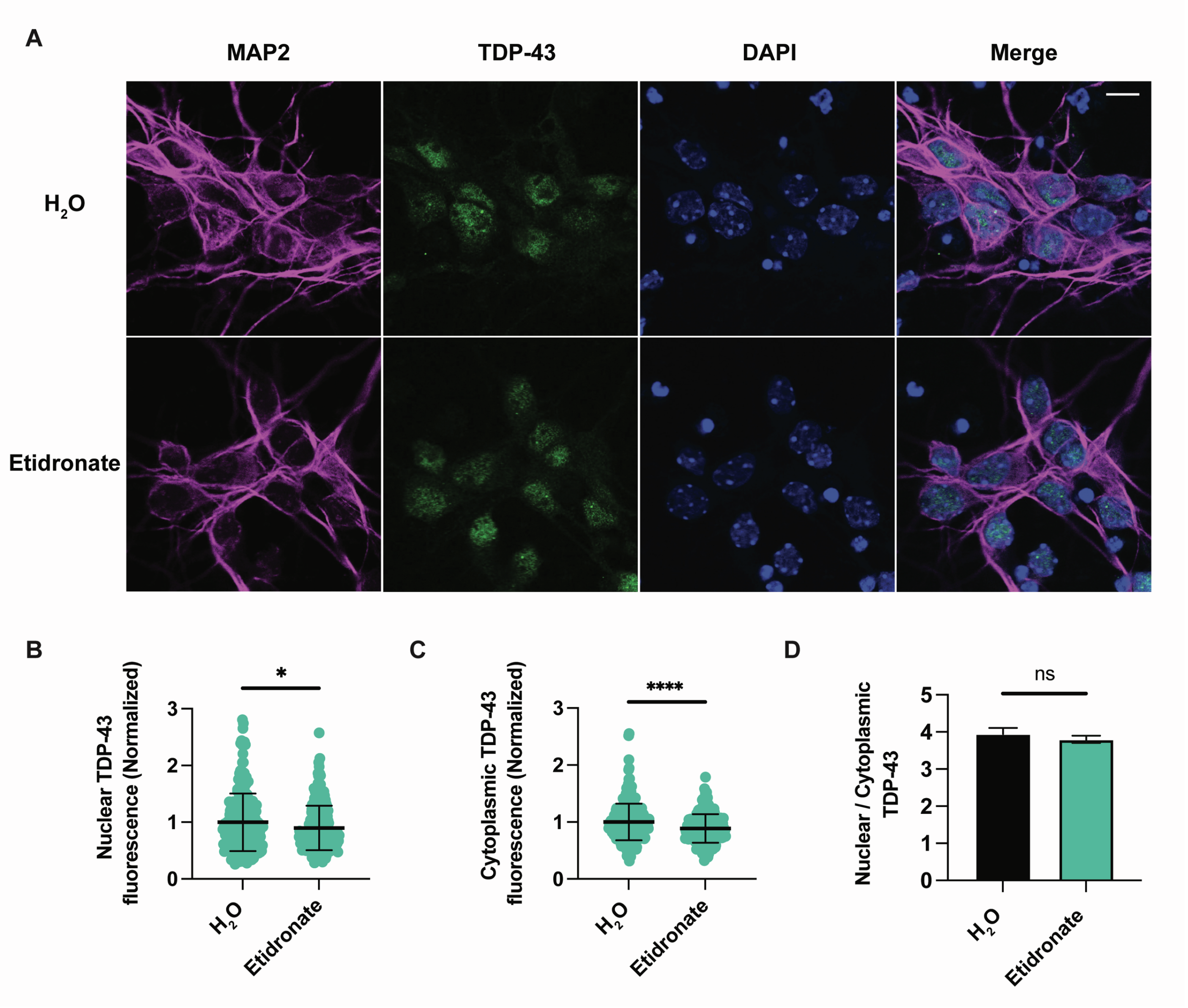

<u>Figure S7: Treating mouse cortical neurons with Etidronate does not affect TDP-43 localization, related to Figure 4</u>. **(A)** Representative microscopy images of mouse primary neurons treated with sham (H_2O) or 10 µM Etidronate for 24 hours then stained for TDP-43, MAP2, and DAPI (scale bar = 10 µm). Quantifications of TDP-43 fluorescence in the nucleus and cytoplasm (normalized to sham condition) are shown in **(B)** and **(C)**, respectively (lines denote mean ± SD; analyzed using unpaired t-test; *: $p<0.05$, ****: $p<0.0001$). **(D)** While TDP-43 fluorescence is very moderately decreased in both the nucleus and cytoplasm, the TDP-43 nucleus-to-cytoplasmic ratio is not affected (mean ± SEM; ns = not significant).
